# Mindset Variations Among Undergraduate Neuroscience Students

**DOI:** 10.59390/001c.154215

**Published:** 2025-12-31

**Authors:** Jade A. Jones, Patrick R. Harrison, Bryant L. Hutson, Sabrina D. Robertson

**Affiliations:** 1 Department of Psychology and Neuroscience University of North Carolina at Chapel Hill; 2 University of North Carolina at Chapel Hill

**Keywords:** growth mindset, fixed mindset, neuroscience education, equity in STEM, historically excluded groups, undergraduate students

## Abstract

Growth mindset, the belief that intelligence is malleable, is linked to academic success and resilience, particularly in STEM disciplines. Prior research suggests that students from historically excluded groups (defined by race, gender, and generation status) often exhibit weaker growth mindsets in STEM before interventions are introduced, primarily due to systemic barriers and heightened stress. We used a quantitative and qualitative approach to assess undergraduate students’ mindsets and compared mindsets across demographic groups. Most mindset studies focus on introductory STEM courses in higher education or K-12 populations. To our knowledge, our study is the first to explore mindsets in an undergraduate neuroscience context. Our findings challenge previous research, revealing that most neuroscience students display growth mindsets without intervention. Notably, growth mindset ratings were significantly higher among students who self-identified as belonging to racial and ethnic minority groups such as PEERs (Persons Excluded because of their Ethnicity or Race). These results highlight the need for further exploration of mindset across diverse demographics, particularly within the context of neuroscience education, as existing research largely focuses on disciplines such as biology, chemistry, mathematics, and physics.

Carol Dweck’s theory of growth mindset presents a transformative perspective on intelligence, proposing that individuals who embrace a growth mindset see intelligence as malleable and often achieve stronger academic outcomes and engage more in challenging tasks. In contrast, a fixed mindset frames intelligence as innate and unchangeable (Dweck, 2006; Richardson et al., 2020).

Studies have identified neurological markers associated with both mindsets, with growth mindsets linked to heightened activity in motivation and reward processing regions (Myers et al., 2016; Ng, 2018). Growth mindsets are also associated with greater psychological resilience and lower cortisol levels, while fixed mindsets correlate with elevated stress responses during academic challenges (Lee et al., 2018; Rege et al., 2021).

When facing academic challenges, many undergraduates struggle to persist, often attributing failure to a lack of intelligence rather than the need for practice and perseverance (Limeri, Carter, et al., 2020). Fixed mindsets are particularly prevalent in STEM fields, despite evidence that growth mindsets can help students navigate difficulties by framing learning as a process independent of prior knowledge or innate ability (Canning & Limeri, 2023). This mindset can discourage students from pursuing specific courses, majors, or careers, with approximately one-third of STEM students leaving the field by the end of their first year (Hansen et al., 2023).

The issue is especially concerning for students from historically excluded groups, such as Black and Latine/Hispanic students, who earn disproportionately fewer STEM degrees, take longer to complete their programs, or fail to graduate altogether. These students face higher rates of academic racism and microaggressions, further exacerbating exclusion (Durkee et al., 2021; Hansen et al., 2023). Research suggests that fixed mindsets disproportionately affect historically excluded groups, potentially contributing to the lack of diversity in STEM fields. However, growth mindsets have been shown to mitigate these challenges by fostering resilience, self-reliance, and persistence in STEM (Richardson et al., 2020; Yeager & Dweck, 2012).

Growth mindset can also strengthen STEM belonging, which is vital for persistence, especially for historically excluded groups (Hansen et al., 2023). Students from underrepresented racial/ethnic groups often lack a sense of community among peers and faculty who do not share their identities, which negatively impacts retention rates (Hansen et al., 2023). Additionally, these students may experience stereotype threat; endorsement of stereotypes and feeling pressure to represent one’s entire race academically can undermine performance and create stronger alignment with fixed mindsets (Wang et al., 2022). Steele and Aronson (1995) demonstrated this effect when Black students performed worse on a challenging verbal test after being told it assessed intrinsic abilities. This phenomenon may extend to standardized tests and STEM exams, perpetuating stereotypes and exclusion (Steele & Aronson, 1995). However, adopting a growth mindset can mitigate stereotype threat. Aronson et al. (2002) found that Black students with a growth mindset reported greater enjoyment in learning, higher engagement, and better academic performance, with a decreased vulnerability to stereotype threat.

These findings have also prompted investigation of growth mindset effects in highly gendered fields like physics. Malespina et al. (2022) found that women were less likely than men to believe they could succeed in calculus-based physics courses. They also increasingly perceived physics as requiring innate ability, which diminished their self-efficacy and contributed to lower retention rates. In biology, Pelch (2018) found that female students were more prone to self-doubt under academic pressure than male peers. Canning et al. (2024) showed that first-generation and low-socioeconomic status (SES) biology students also face self-efficacy challenges reducing persistence, but growth mindset interventions improved grades for all students, particularly benefiting first-generation students. Chemistry studies reveal similar mindset patterns across diverse populations. Limeri et al. (2020) found that students struggling academically in organic chemistry were more likely to adopt fixed mindsets. However, Fink et al. (2018) demonstrated that growth mindset interventions tied to exams helped narrow racial achievement gaps between white students and those from underrepresented backgrounds. Gender disparities also emerge, with Wheeler and Gonczi (2022) finding that women’s self-efficacy in chemistry may decline as negative lab experiences reduce engagement.

Although mindset trends are evident across physics, chemistry, and biology, no previous studies have examined mindsets in neuroscience undergraduates. Neuroscience’s interdisciplinary nature, with its focus on neuroplasticity and the brain’s capacity for change, may foster growth mindsets (Limeri, Carter, et al., 2020) and unique variations across groups. Our study addresses this gap by investigating how mindsets vary among neuroscience students at a large R1 public university, with particular emphasis on students from diverse demographics underrepresented in STEM. Using a mixed-methods design, we combined quantitative survey data with qualitative analysis to capture mindset trends. Based on the research described above in related disciplines (chemistry, physics, and biology), we hypothesized that individuals from historically excluded groups would exhibit lower growth mindsets in the absence of formal interventions. However, Latine/Hispanic students demonstrated significantly higher growth mindsets compared to their peers. These findings challenge prevailing assumptions about mindset disparities among historically excluded students. Notably, they suggest how disciplinary context, particularly neuroscience curricula’s focus on neuroplasticity and growth mindset, may shape mindset development. Our results demonstrate the need to expand mindset research to include neuroscience contexts, encouraging the development of more effective, equity-centered interventions.

## MATERIALS AND METHODS

### Participants

Participants (*N* = 309) were recruited from undergraduate neuroscience courses at a large public R1 institution. Participants were recruited from 100, 200, 300 level neuroscience courses during the Spring and Summer 2024. Participation was voluntary, and all participants provided consent. Of the students who submitted the survey, 255 completed all quantitative questions (response rate = 82.5%), and 254 provided substantive responses to the qualitative questions (response rate = 82.2%).

Among respondents, 252 reported their racial or ethnic identity with 26.0% identified as belonging to historically excluded racial or ethnic groups, referred to as Persons Excluded due to Ethnicity or Race (PEERs) (Asai, 2020). We adopted the term PEERs to shift focus from framing underrepresentation as a trait of marginalized groups to highlighting systemic barriers in science and education (Asai, 2020). In this study, the PEERs category encompassed four subgroups: Black, African American, Afro-Caribbean (*n* = 17); Latine/Hispanic American (*n* = 27); Middle Eastern/Arab American (*n* = 6); and Multiracial individuals (*n* = 17).

In terms of generational status, 74.5% of respondents were continuing-generation students, while 25.5% were first-generation students. Gender representation included 60.0% cisgender women, 32.9% cisgender men, and 7.1% who identified as non-conforming or preferred not to disclose. Participants’ ages ranged from 18 to 40 years, with a mean age of 20.7 years. Table 1 summarizes the demographic characteristics of the final participant sample.

**Table 1.** Participant Demographics

**Descriptor**	**Number of respondents (% of respondents)**
**Gender**	*255 participants reported their gender identity*
Cisgender Man	84 (32.9%)
Cisgender Woman	153 (60.0%)
Non-conforming	18 (7.1%)
**Racial/Ethnic Identity**	*252 participants reported their racial/ethnic identity*
White, Non-Hispanic	93 (36.9%)
Latine / Hispanic American	27 (10.7%)
Black, African American, Afro-Caribbean	17 (6.8%)
East Asian / Asian American	41 (16.3%)
South Asian / Indian American	52 (20.6%)
Middle Eastern / Arab American	6 (2.4%)
Multiracial	16 (6.4%)
**Generation in College**	*255 participants reported their generation status*
First-Generation	65 (25.5%)
Continuing-Generation	190 (74.5%)

### Survey Instrument

An online survey was developed to assess students’ mindset perceptions in neuroscience and STEM fields using previously validated measures (see Supplementary Table 1). The survey explored six categories: (1) Defining Intelligence and Failure, (2) Growth Mindset, (3) Identity, Intelligence, and Stereotype Threat, (4) Instructor Trust and Beliefs about Instructor Mindset, (5) Persistence, and (6) Demographics. We focus on Defining Intelligence and Failure (free-response questions – Limeri, Choe, et al., 2020), Growth Mindset (Likert scale – Dweck, 2006; Malespina et al., 2022), and Demographics.

Administered via Qualtrics, the survey included both quantitative and qualitative questions. Most quantitative items related to growth mindset were negatively coded and used a 5-point Likert scale (1 = Strongly Disagree to 5 = Strongly Agree). Responses to quantitative items were visualized using color-coded bars: dark blue for “strongly disagree” (1), light blue for “disagree” (2), gray for “neither agree nor disagree” (3), orange for “agree” (4), and red for “strongly agree” (5. Stronger disagreement indicates a higher growth mindset, while stronger agreement indicates a higher fixed mindset. For some qualitative questions, participants selected “yes” or “no” before providing detailed open-ended responses.

### Data Collection

This study was reviewed and approved by the Institutional Review Board (IRB# 23-2891). All participants were informed of their right to withdraw at any time and assured of confidentiality. Data collection occurred from April 2024 to July 2024. Neuroscience instructors introduced the survey in their courses and offered extra credit as an incentive. To mitigate coercion, students were given an alternative extra credit opportunity unrelated to the survey, capped at no more than 1% of the final course grade. Participants accessed the survey through an anonymous link but included their student ID numbers (PIDs) to verify participation. Data was de-identified as student IDs were replaced by ordered participant markers (i.e., P1). Electronic consent was obtained before beginning the survey. The survey took approximately 15 minutes to complete.

### Quantitative Data Analysis

The quantitative analysis followed a multi-step protocol to ensure accurate and reproducible results. Initial data processing began with organizing raw survey responses in Microsoft Excel (v16.95), where each participant’s unique marker was maintained adjacent to their demographic identifiers (e.g., race/ethnicity, gender identity, major, generation status, or academic year) and all eight responses to the set of mindset questions (5-point Likert scale: 1 = Strongly Disagree to 5 = Strongly Agree).

For each demographic identifier, we created subgroup-specific data tables by partitioning responses according to self-reported identities. For instance, gender data was separated into three distinct tables containing: all responses from cisgender women, all responses from cisgender men, and all responses from non-conforming individuals. Similarly, racial/ethnic data was partitioned–first into broad categories (PEERs, White, Asian), then refined into subgroup tables (e.g., PEERs → Black, Latine/Hispanic, etc.; Asian → East Asian, South Asian). Microsoft Copilot AI efficiently assisted in automating table creation with consistent formatting. Copilot applied predefined demographic categorization keys to participant markers and quantitative data while ensuring consistent subgroup isolation across all analyses.

**Figure 1.**
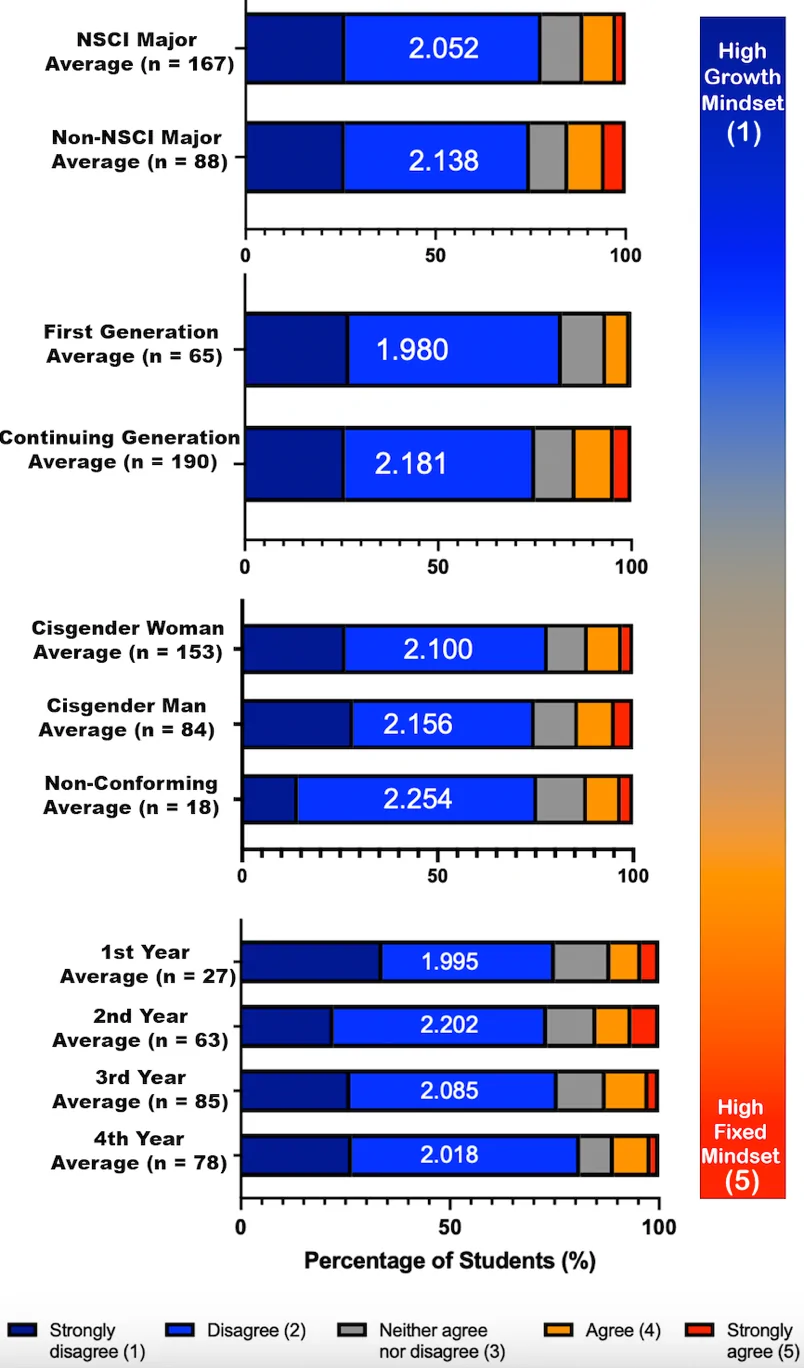
Average Mindset Responses Across Diverse Demographic Groups. Graphs display composite mindset scores. Groups are categorized by major (Neuroscience [NSCI] Major vs. Non-NSCI Major), gender identity (Cisgender Woman, Cisgender Man, and Non-Conforming), generation status (First-Generation vs. Continuing-Generation), and academic year (1st, 2nd, 3rd, and 4th year). Average mindset scores are displayed within each bar, with higher mindset scores (closer to 5) indicating stronger agreement with fixed mindset statements and lower scores (closer to 1) reflecting stronger agreement with growth mindset statements. Sample sizes for each group are provided on the figure (*n* = sample size). Statistical analyses included Mann-Whitney U tests for major and generation status groups, which yielded no significant differences (Major: *p* = 0.504; Generation: *p* = 0.168). One-way ANOVAs were conducted for gender identity and academic year, with no significant differences detected between groups, as determined by Tukey’s post hoc analysis (Gender: adjusted *p* = 0.657; Academic Year: adjusted *p* = 0.356).

**Figure 2.**
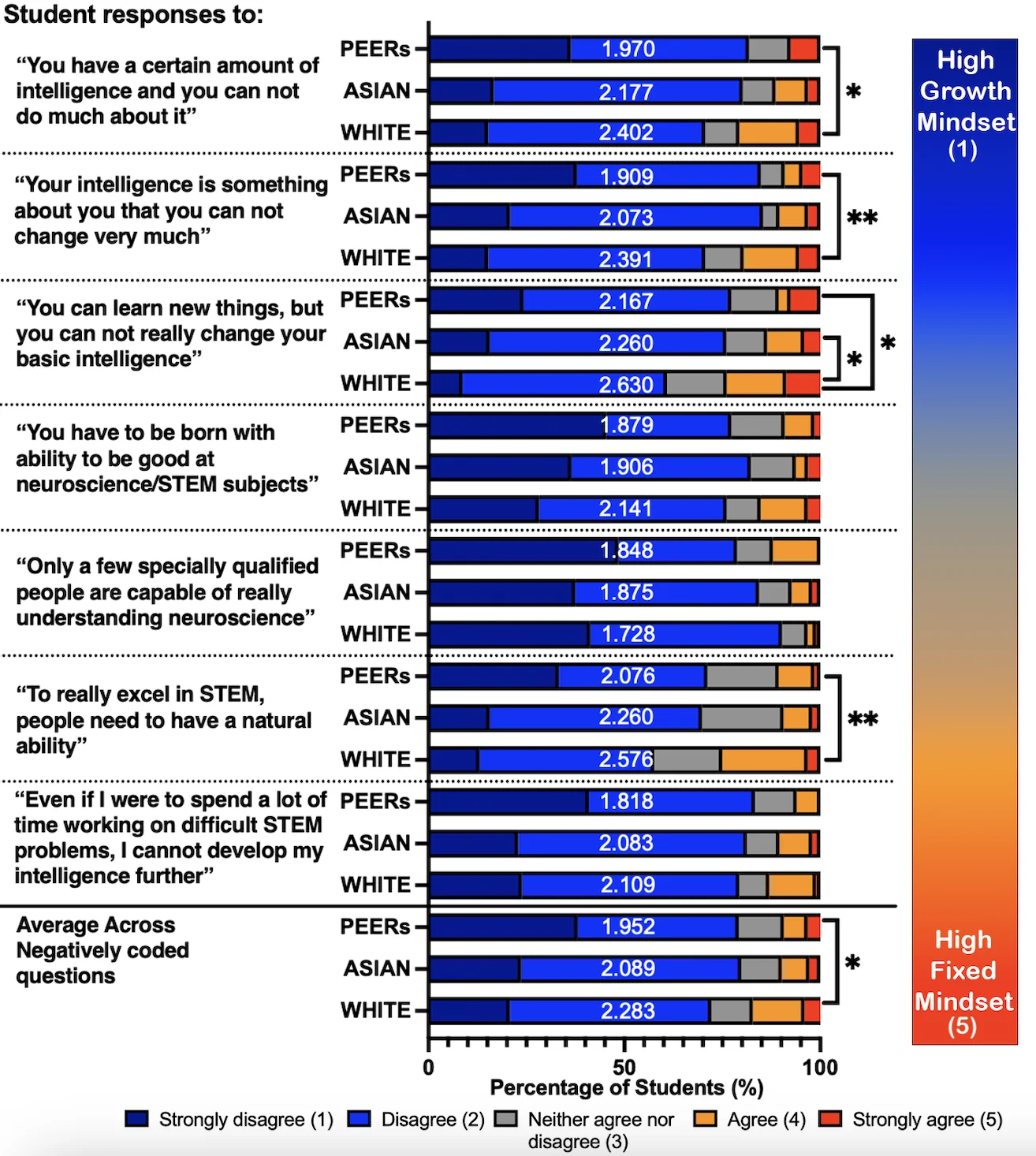
Student Mindsets Differ Across Racial and Ethnic Groups in Neuroscience. The bar graphs display composite mindset scores across three racial groups: PEER (*n* = 66), Asian (*n* = 96), and White (*n* = 92). The average mindset score for each group is displayed within each bar, with higher scores (closer to 5) indicating stronger agreement with fixed mindset statements while lower scores (closer to 1) reflect stronger agreement with growth mindset statements. A one-way ANOVA revealed a statistically significant difference between the averages across questions for PEERs (*M* = 1.95) and White participants (*M* = 2.28) with a p = 0.016, adjusted using Tukey’s post hoc analysis. Additional individual questions showed statistical significance (“You have a certain amount of intelligence and you cannot do much about it”: adjusted *p = 0.026; “Your intelligence is something about you that you cannot change very much”: *p = 0.009; “You can learn new things, but you cannot really change your basic intelligence”: *p = 0.019, **p = 0.044; “To really excel in STEM, people need to have a natural ability”: **p = 0.005).

**Figure 3.**
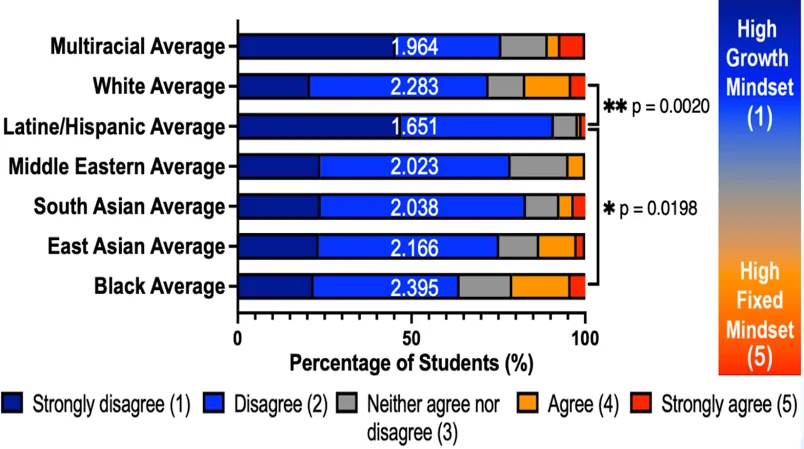
Student Mindsets Differ Across Racial and Ethnic Subgroups in Neuroscience. The graphs display composite mindset scores across six subgroups: Black, African American, Afro-Caribbean (*n* = 17), East Asian/Asian American (*n* = 41), South Asian/Indian American (*n* = 52), White, Non-Hispanic/European American (*n* = 92), Latine/Hispanic American (*n* = 27), Middle Eastern/Arab American (*n* = 6), and Multiracial (*n* = 16). The average mindset score for each group is displayed within each bar, with higher scores (closer to 5) indicating stronger agreement with fixed mindset statements and lower scores (closer to 1) reflecting stronger agreement with growth mindset statements. A one-way ANOVA revealed statistically significant differences between Latine/Hispanic participants (*M* = 1.65) and White participants (*M* = 2.28), with an adjusted p-value of 0.002, as determined by Tukey’s post hoc analysis. Additionally, a statistically significant difference was observed between Black participants (*M* = 2.40) and Latine/Hispanic participants (*M* = 1.65), with an adjusted p-value of 0.019.

Three analytical approaches were implemented: individual-level analysis calculated each participant’s average mindset score across the mindset questions; item-level analysis computed the distribution of responses for each question within demographic groups by summing absolute counts for each Likert response, then converting these to percentages by dividing by the group’s total respondent count; and finally, composite analysis averaged the item-level response distributions across questions to produce the overall average for a demographic group and averaged the individual-level participant means to produce the composite mindset score (used to create Figures 1–3).

Quality control measures included automated validation in Excel to verify that participant totals matched across analyses; manual spot-checking of random samples to confirm accurate demographic categorization within tables; and cross-verification of all composite calculations with the descriptive statistics feature in GraphPad Prism (v10.4.1).

### Qualitative Data Analysis

The qualitative investigation of open-ended responses sought to identify and characterize specific factors influencing students’ mindsets (Supplementary Table 1). To qualitatively analyze students’ responses, deductive and inductive coding methods were modeled after Gin et al. (2018) and Limeri et al. (2020).

**Figure 4.**
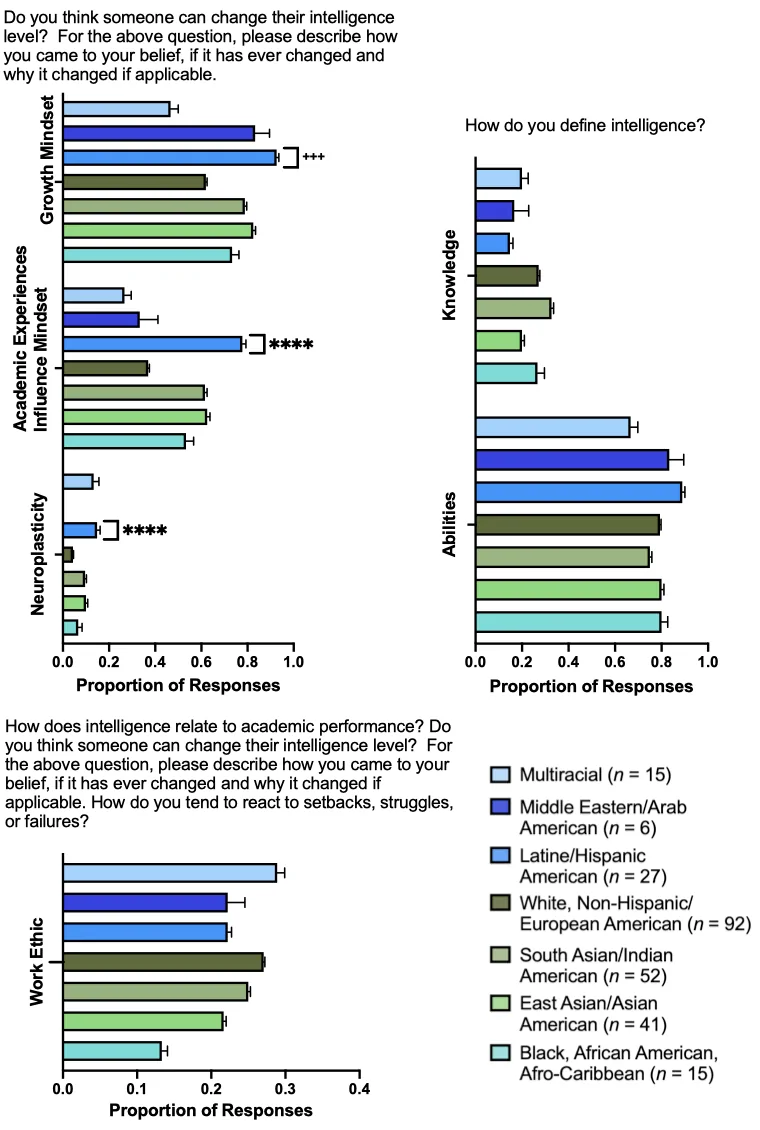
Mindset Themes Differ across Racial and Ethnic Subgroups in Neuroscience. Students responded to several qualitative questions exploring their definitions of intelligence, the relationship between intelligence and academic performance, beliefs about whether intelligence can change, and reactions to struggles or failure (questions in Supplemental Table 1). Responses were qualitatively coded for the presence of six key themes: Growth Mindset, Academic Experiences Influencing Mindset, Neuroplasticity, Knowledge, Abilities, and Work Ethic. Coding was based on both direct mentions and interpretation of the students’ responses. A quantitative comparison of the proportion of students whose responses included each theme was conducted across racial and ethnic subgroups to expand on quantitative findings (total *N* = 247 students, total excluding respondents that did not list their race): Black, African American, Afro-Caribbean (*n* = 15), East Asian/Asian American (*n* = 40), South Asian/Indian American (*n* = 52), White, Non-Hispanic/European American (*n* = 92), Latine/Hispanic American (*n* = 27), Middle Eastern/Arab American (*n* = 6), and Multiracial (*n* = 15). Only select statistical findings are included in this figure. All statistical analyses are included in Supplemental Tables 3-8. The +++ symbol indicates that Latine/Hispanic students’ proportion of growth mindset responses were significantly higher than all other groups (p < 0.0001) except Middle Eastern/Arab Americans (p <0.05) (see Supplemental Table 3) . For Academic Experiences (Supplemental Table 4) and Neuroplasticity (Supplemental Table 5), Latine/Hispanic students differed from all groups at p < 0.0001****. Error bars represent the standard error of the mean.

The deductive coding methodology followed a structured approach, applying thematic codes to open-ended responses regardless of quantitative findings or emerging qualitative themes (Gin et al., 2018). These predefined codes were derived from established constructs of interest, including growth mindset, neuroplasticity, and knowledge versus abilities which were applied to all relevant responses addressing mindset and intelligence beliefs (see figure 4) (Limeri, Choe, et al., 2020). Consistent with the protocol, these codes functioned as standardized descriptors with operational definitions referenced throughout reading, coding, and recoding (Gin et al., 2018).

For the inductive coding approach, open-ended responses were reviewed iteratively to identify frequently referenced codes (Limeri, Carter, et al., 2020). After an initial review, commonly referenced themes outside of the established deductive codes were compiled, and responses were reanalyzed to ensure no references were missed (Limeri, Carter, et al., 2020). Key inductive codes included work ethic principles and academic experiences (figure 4).

The qualitative data were coded, and then the numerical data were analyzed. First, the frequency of each code within participants’ free-response answers was tallied, with responses grouped by the self-reported racial/ethnic identity. These raw counts were then converted to proportional values, as each code count was divided by the total number of responses from the corresponding demographic group. The standard error of the mean was computed for the proportion of responses. Between-group comparisons were performed using one-way ANOVAs in GraphPad Prism (v10.4.1) to identify statistically significant differences in code prevalence across racial/ethnic categories, enabling quantitative evaluation of how mindset patterns varied demographically (Figure 4).

Intercoder reliability was evaluated using Microsoft Copilot AI (see Supplemental Materials for prompts - Supplementary Table 2), with Cohen’s κ values calculated for each code, indicating agreement levels between coders: Neuroplasticity (κ = 0.72, substantial), Work Ethic (κ = 0.64, substantial), Growth Mindset (κ = 0.47, moderate), Abilities (κ = 0.28, fair), Knowledge (κ = 0.21, fair), and Academic Experiences (κ = 0.15, slight). The researcher compared AI-generated and human-coded responses in Excel, resolving discrepancies through manual review (Limeri, Carter, et al., 2020). Due to inconsistencies between Copilot and human coding, final quantification (Figure 4) prioritized human judgments. Valid AI-identified codes that aligned with operational definitions were incorporated when missed by the researcher. While Copilot performed well on straightforward responses, complex, nuanced, and ambiguous responses exceeded Copilot’s capabilities and required human intervention despite coaching attempts and emphasizing code definitions. All final codes and their operational definitions are provided (Table 2 and Supplemental Table 9 for additional inductive codes).

### Statistics

Quantitative data were analyzed using Microsoft Excel (v16.95) and GraphPad Prism (v10.4.1). Descriptive statistics (means, standard deviations, standard errors, and percentages) were computed for all mindset measures. For demographic comparisons between two subgroups (neuroscience majors vs. non-majors; first-generation vs. continuing-generation students), we employed Mann-Whitney U tests with α = 0.05. For analyses involving three or more subgroups (gender identity: cisgender women, cisgender men, and non-conforming individuals; race/ethnicity: PEERs, Asian, and White; and detailed racial subgroups: Black/African American/Afro-Caribbean, Latine/Hispanic American, Middle Eastern/Arab American, Multiracial, East Asian/Asian American, South Asian/Indian American, and White/Non-Hispanic/European American), we conducted one-way ANOVAs followed by Tukey’s post hoc tests to identify specific between-group differences in composite mindset scores. Qualitative code frequencies were quantified as proportions of respondents referencing each theme (with standard errors), and one-way ANOVAs compared these proportions across racial/ethnic subgroups to assess alignment with quantitative mindset results. All conclusions drawn with the statistical approaches detailed here (one-way ANOVA) were also confirmed with the use of non-parametric statistics (Kruskal-Wallis tests and Dwass-Steel-Critchlow-Fligner post hoc analysis).

**Table 2.** Deductive Codes, Inductive Codes, and Code Definitions.

**Code Type**	**Code**	**Definition**
**Deductive**	Growth Mindset	Characterized by responses indicating intelligence is malleable and can be developed through effort, learning, or education (Dweck, 2006).
	Fixed Mindset	Characterized by responses suggesting intelligence as an innate quality with predetermined capacity (Dweck, 2006).
	Knowledge	Refers to defining intelligence as one's current level of knowledge, independent of effort or time invested in gaining it (Limeri, Choe, et al., 2020).
	Abilities	Characterized by viewing intelligence as what one can do with their knowledge (e.g., learning, problem-solving, applying knowledge to new contexts) (Limeri, Choe, et al., 2020).
	Neuroplasticity	Defined by references to brain reorganization, neural connectivity adaptation, and the brain's lifelong capacity for change (Ng, 2018).
**Inductive**	Work Ethic	Emphasizes effort dedicated to tasks as the primary driver of achievement (Dweck & Yeager, 2019).
	Academic Experiences	Educational events that shaped mindset beliefs throughout one's life (Yeager & Dweck, 2012).

## RESULTS

### Comparison of Students’ Mindsets across Major, Generation Status, Gender, and Academic Year

To determine the mindset variations that existed among neuroscience students, mindset scores across major, generation status, gender identity, race/ethnicity, and academic year were averaged. There were no statistically significant differences within major, gender identity, generation status, or academic year. However, key observations can still inform future research (Figure 1).

### Major

Neuroscience majors (*M* = 2.05) and non-neuroscience majors (*M* = 2.14) exhibited scores consistent with a growth mindset and they were not significantly different (*p* = 0.504). All students reported average scores below 3, reflecting general alignment with growth mindset principles regardless of major. It is important to note that every non-major student in our sample was enrolled in a neuroscience course and may have taken additional neuroscience courses previously.

### Generation Status

Contrary to literature suggesting first-generation students exhibit weaker growth mindsets in STEM, first-generation students demonstrated comparable mindset scores (*M* = 1.98) to continuing-generation students (*M* = 2.18). The difference was not statistically significant (*p* = 0.168). This finding is interesting given that first-generation students often benefit most from explicit mindset interventions (Canning et al., 2019, 2024; Hansen et al., 2023; Hecht et al., 2022; Myers-Miller, 2024). Our results suggest that neuroscience students may naturally cultivate growth beliefs, perhaps through their courses’ emphasis on neuroplasticity and adaptable intelligence, mirroring the effects of formal mindset interventions. Future studies should compare first-generation neuroscience students with their peers in other STEM disciplines.

### Gender Identity

In contrast to literature indicating women gravitate toward fixed mindsets in challenging STEM courses (Malespina et al., 2022), cisgender women show growth mindsets (*M* = 2.10). Additionally, cisgender men (*M* = 2.15) and non-conforming students (*M* = 2.25) exhibit scores aligning with growth mindset. No significant differences emerged between groups. The female-majority composition (60.0%) may reduce vulnerability to fixed mindsets compared to male-dominated STEM fields. (Myers-Miller, 2024).

### Academic Year

First-year (*M* = 1.995), second year (*M* = 2.20), third year (*M* = 2.09) and fourth year (*M* = 2.02) students showed scores consistent with growth mindsets, and there were no significant differences between groups. Based on the neuroscience curriculum at the institution, it is common for neuroscience majors to take computer science, physics, chemistry, and biology courses during their second and third years. Mindset trends may align more with those disciplines within second- and third-year students, potentially reflecting the broader STEM context rather than neuroscience-specific experiences. We also found no significant correlation between the number of neuroscience courses taken and mindset scores (Spearman’s r = −0.08, p = 0.202).

### Neuroscience Student Mindsets vary significantly between Racial and Ethnic Groups

We next explored how mindsets differed among racial and ethnic groups. We compared PEERs, Asian, and White students’ responses. Previous studies suggest that historically excluded groups often experience additional stress and systemic disadvantages due to discriminatory structures, which can influence mindsets and hinder participation in STEM (Akos et al., 2020; Burnette et al., 2022; Durkee et al., 2021). Since no interventions were implemented in this study, we expected historically excluded students to exhibit significantly lower growth mindsets. However, our results revealed the opposite: PEERs students demonstrated the same or even higher baseline growth mindsets compared to their Asian and White peers (Figure 2). The PEERs group had a mindset score (*M* = 1.95), significantly lower than that of White students (*M* = 2.28), indicating a stronger growth mindset among PEERs. There was no significant difference between Asian students (M = 2.09) and either PEERs or White students. The PEERs group’s lower mean suggests they are more likely to reject fixed mindset beliefs and embrace growth mindset beliefs. While PEERs, White, and Asian students’ responses varied, all mindset scores fell below 3, indicating that all groups aligned more with growth mindset statements.

These findings differ from prior research suggesting that students from historically excluded STEM groups, such as PEERs, often have weaker growth mindsets due to stereotype threat (Canning et al., 2019; Limeri, Carter, et al., 2020). Instead, our results suggest neuroscience education may uniquely shape student mindsets. But it remains unclear whether these differences existed before entering the curriculum or were influenced by the academic environment. Further research is needed to determine if neuroscience education or other external factors contribute to these mindset differences among racial and ethnic groups.

After examining mindset variations within overarching racial groups (Figure 2), we next compared all racial and ethnic subgroups. We compared mindset scores across Black/African American/Afro-Caribbean, Latine/Hispanic American, Middle Eastern/Arab American, Multiracial, East Asian/Asian American, South Asian/Indian American, and White/Non-Hispanic/European American students. We found the Latine/Hispanic group had a mindset score of 1.651, significantly lower than White participants (*M* = 2.28; adjusted p-value = 0.002, as determined by Tukey’s post hoc analysis). This indicates that Latine/Hispanic students demonstrated a stronger growth mindset compared to their White peers and drove the statistical significance observed within the PEERs group. This may reflect cultural values, educational experiences, or other factors that emphasize adaptability and resilience.

Additionally, a significant difference was observed between Black and Latine/Hispanic participants. Black participants had an average mindset score of 2.395, reflecting a weaker growth mindset compared to Latine/Hispanic participants (*M* = 1.65; adjusted *p*-value = 0.0198). However, both groups’ average scores remained below 3, indicating an overall tendency toward growth mindset beliefs. The difference between Black and Latine/Hispanic participants show that mindsets do not manifest the same within subgroups under the PEERs umbrella.

Figure 3 also demonstrates the diversity within the PEERs and Asian categories. South Asian and East Asian students showed moderate mindset scores, with no significant differences between them. Therefore, despite the shared history of exclusion from STEM among groups, mindset trends must be examined within subgroups rather than treating racial and ethnic categories as monolithic.

### Analysis of Students’ Beliefs about Mindset across Racial and Ethnic Groups

Following our quantitative findings that PEERs (particularly Latine/Hispanic students) exhibited stronger growth mindsets than their Asian and White peers (Figures 2 and 3), we analyzed qualitative responses to understand how students conceptualized intelligence. We coded open-ended responses from 254 participants using both deductive (growth mindset, neuroplasticity, knowledge vs. abilities) and inductive (work ethic, academic experiences) themes (6 total in Figure 4) and highlighted key differences in mindset-related themes across racial/ethnic subgroups. The themes reveal how students perceive intelligence and the factors shaping their beliefs.

Growth mindset coding was characterized by responses indicating intelligence could be developed through effort, learning, or education (Dweck, 2006). Fixed mindset coding was characterized by responses suggesting intelligence as an innate quality with predetermined capacity (Dweck, 2006). Academic experiences were characterized by educational events that shaped mindset beliefs throughout one’s life (Yeager & Dweck, 2012).

Neuroplasticity was defined by references to brain reorganization, neural connectivity adaptation and development, and the brain’s lifelong capacity for change (Ng, 2018).The knowledge code was characterized by defining intelligence as one’s current level of knowledge, independent of the effort or time invested in gaining it (Limeri, Choe, et al., 2020). Abilities were characterized by viewing intelligence as what one can do with their knowledge, including learning, problem-solving, applying knowledge to new contexts, and critical thinking (Limeri, Choe, et al., 2020). Lastly, work ethic was characterized by emphasizing effort dedicated to tasks as the primary driver of achievement (Dweck & Yeager, 2019).

### Growth versus Fixed Mindset

The quantitative data showed the strongest alignment with growth mindset for the Latine/Hispanic group, and the qualitative data further supported this finding, as growth mindset was coded in 93% of Latine/Hispanic American responses. Although certain subgroups referenced growth mindset more frequently, every subgroup referenced the concept and provided responses supporting the theory.

“I have read a lot of papers and wrote my manuscript on growth mindset and resilience. Both my research and the existing literature confirmed my belief that intelligence is malleable. This is mainly based around the idea of the theories of intelligence that I spent a lot of time learning about for my research.” (White, Non-Hispanic/European American)

“I do not believe that anyone ever has a set intelligence level and they can always learn new things” (Latine/Hispanic American)

“No one has a fixed level of intelligence; you can always change the way you learn and develop your knowledge through practice.” (White, Non-Hispanic/European American)

“I don’t think I used to think of intelligence as changeable because of genetic factors that are shown in IQ tests. However, there’s been a lot more research of the malleable nature of the brain and change in how much information can be retained and how it can be used.” (South Asian/Indian American)

“There is evidence that we can all grow a growth mindset instead of a fixed one. Even from a cellular level, our brain plasticity allows us to produce neurons, especially after experiencing brain trauma.” (Latine/Hispanic American)

Qualitative responses revealed strong alignment with growth mindset principles, with many participants explicitly stating their belief in the malleability of intelligence. Several students referenced academic or scientific evidence to support their views. For example, a student cited their research on growth mindset and resilience, emphasizing theories of intelligence that affirm its malleability. Similarly, two students both expressed the belief that intelligence is not fixed and can be developed through learning and practice. Some participants also highlighted scientific evidence, such as brain plasticity, to support their views. One student noted a shift in their perspective, acknowledging recent research on the brain’s malleability. Additionally, another student referenced neuroplasticity and neurogenesis as evidence supporting growth mindset. These responses demonstrate how students’ beliefs about intelligence are often informed by both personal experience and buy-in to the science.

### Fixed Mindset

While most participants expressed growth mindset beliefs (73%), a few responses reflected fixed mindset perspectives (15%).

“I used to believe that intelligence was based on how hard you worked. However, I have realized that intelligence is often determined at a young age and is rather inflexible once older.” (White, Non-Hispanic/European American)

“People cannot change their intelligence, but they can work around to compensate for it.” (East Asian / Asian American)

For example, one student described a shift in their belief, stating that intelligence is largely determined at a young age, regardless of work ethic, and becomes inflexible over time. Similarly, another student acknowledged that while intelligence cannot be changed, individuals can work around their weaknesses to compensate for lacking ability at times. These responses highlight the persistence of fixed mindset beliefs among some students, even within a population that largely aligns with growth mindset principles.

### Academic Experiences Shaping Mindset

We next examined how academic experiences influenced students’ beliefs, as educational contexts play a critical role in reinforcing or reshaping mindsets (Dweck & Yeager, 2019). Within STEM disciplines, research demonstrates that academic struggles often trigger fixed mindsets when students lack proper support (Cohen & Garcia, 2005; Limeri, Carter, et al., 2020). However, when educators intentionally structure challenges to emphasize growth and learning processes, these same difficulties can become opportunities to build resilience and strengthen growth mindsets (Dweck & Yeager, 2019). Our inductive coding revealed that 50% of participants explicitly connected their mindset development to academic experiences. Students’ accounts consistently fell into two categories: educational opportunities provided by their institution or personal experiences of academic struggle.

“I think that I have become more intelligent since coming to UNC. The more educated you become, the more intelligent you are, as you learn information and how to apply it.” (Latine / Hispanic American)

“I used to have low test scores and struggled in high school, but when I learned to apply myself and focus, I began to succeed academically.” (Latine / Hispanic American)

“Starting school in America, knowing no English, I worked hard to understand concepts. My belief has never changed: with the right motivation and perseverance, intelligence can change.” (East Asian / Asian American)

For instance, one student attributed increased intelligence to their college education, while another student described overcoming academic struggles through effort and focus. The final student shared how their experience learning English in the U.S. reinforced their belief in the malleability of intelligence through motivation and perseverance. Based on prior literature, we did not expect STEM students to leverage their academic experiences toward growth mindsets; however, neuroscience students differ, as challenging academic experiences do not seem to manifest in a fixed mindset within our student population.

### Neuroplasticity

We next investigated students’ references to neuroplasticity, as neuroscience curricula uniquely emphasize brain malleability–a potential disciplinary advantage for growth mindset development (Ng, 2018). We identified explicit mentions of ‘brain plasticity’ or ‘neuroplasticity,’ as well as descriptions of changing neural connections. However, neuroplasticity was referenced less frequently than expected, appearing in only 8% of total respondents.

“Neuroplasticity means that our brains are not fixed but can grow.” (Latine / Hispanic American)

“I believe intelligence can change because research in neuroscience and psychology shows that the brain is plastic and capable of growth.” (Central Asian)

“I believe intelligence can change due to research on neuroplasticity, which shows that the brain can form new connections and improve function with practice and learning.” (White, Non-Hispanic/European American)

Some students explicitly referenced scientific principles of neuroplasticity as a basis for believing in intelligence’s malleability. Since neuroplasticity is extensively covered in the neuroscience curriculum, we expected more frequent references. However, neuroplasticity mentions remained rare overall yet appeared most frequently among PEERs, particularly Latine students (15%). This pattern contrasts with STEM-wide trends where PEERs typically reference growth mindset concepts less often (Limeri, Choe, et al., 2020). The higher neuroplasticity mention rate among Latine/Hispanic students aligns with their strong growth mindset, suggesting that the curriculum may influence their perspectives, as students referenced specific research and neuroscience terminology when discussing mindset.

### Knowledge or Abilities

Students’ responses to ‘How do you define intelligence?’ (Limeri, Choe, et al., 2020) revealed key findings regarding the discrepancy between defining intelligence as accumulated knowledge versus the ability to acquire and apply knowledge effectively. Prior literature suggests that individuals who define intelligence as knowledge tend to resonate more with fixed mindset ideas, whereas those who define it in terms of abilities align more with growth mindset principles (Limeri, Choe, et al., 2020). As shown in Figure 4, our results support this trend. While participants varied in their growth mindset strength, all subgroups had average mindset scores below 3. This trend is further supported by our knowledge versus abilities coding, as all participant groups referenced abilities more frequently than knowledge. However, 24% of students still defined intelligence primarily as the knowledge one possesses, framing it as the accumulation of facts and information that you either know or do not know.

“I define intelligence as the amount of knowledge or capacity for knowledge that a person possesses.” (Black, African American, Afro-Caribbean)

“Intelligence is the information you know as a person regarding other topics.” (South Asian/Indian American)

The majority of students viewed intelligence as a skill set or an ability that can grow and develop over time, emphasizing adaptability, problem-solving, and creativity (77%).

“Intelligence is the ability to recall previously learned information and know how to use it in proper contexts.” (Latine / Hispanic American)

“True intelligence involves applying knowledge creatively and effectively, not just in conventional ways, but also in innovative and practical scenarios where it truly matters.” (Latine / Hispanic American)

### Work Ethic

While existing literature often cites work ethic as a core component of a growth mindset, many of our respondents expressed the belief that intelligence is fixed but that hard work can compensate for lacking natural ability.

“I think some people are made with greater academic or intellectual ability than others. People can work hard to change how smart they are, but their intelligence cannot change. Or perhaps it can change slightly, but not as much as smartness can.”

This perspective appears somewhat contradictory to the concept of a growth mindset. While they acknowledge that effort can lead to improvement, they distinguish between “smartness” and “intelligence”, suggesting that intelligence itself is unchangeable. This distinction raises questions about how students conceptualize intelligence and effort, and whether the belief in the power of hard work necessarily equates to a fully developed growth mindset. Prior research has been wary of emphasis on effort alone. Focusing exclusively on effort can be counterproductive without emphasizing the efficacy of strategies, resources, support, and mentorship. Students may blame themselves or their lack of effort for their failures or academic shortcomings (Burnette et al., 2022; Dweck & Yeager, 2019). Many other students associated intelligence with discipline, effort, and work ethic.

“…While someone can be very academically intelligent, that doesn’t always mean they are knowledgeable or proficient in that topic. Hard work beats talent when talent doesn’t work hard.” (South Asian/Indian American)

“i think that intelligence isn’t innate so if you work toward excelling in a topic or skill then you’re obtaining the intelligence to master it.” (Latine/Hispanic American)

“I have never thought of myself as academically intelligent, but I believe I have achieved academic success through hard work, dedication, and good habits.” (White, Non-Hispanic / European American)

“Old Chinese saying says the ability on something will be better when it is more frequently trained. This is true for me because I think human’s brain are far from most efficiently used or fully ‘exploited’.” (East Asian / Asian American)

Many students emphasized that hard work and dedication often outweigh innate talent, reflecting a strong alignment with growth mindset principles. For example, one student noted that hard work can surpass talent when talent is not applied. Similarly, students expressed the belief that intelligence is not innate and can be developed through effort and practice. These responses support the idea that effort and persistence are key to achieving mastery, regardless of perceived innate ability.

Other students shared personal experiences that reinforced the value of work ethic. One student explained that their academic success was achieved through hard work, dedication, and good habits, rather than innate intelligence. This perspective highlights how students who may not identify as “naturally intelligent” still attribute their achievements to consistent effort and discipline that any person has the capability to demonstrate. Additionally, another student referenced a cultural proverb to support the view that abilities improve with practice, suggesting that human potential and the brain’s capabilities are often underutilized. This response not only reflects a growth-oriented perspective but also illustrates how cultural values can shape beliefs about intelligence and effort.

### Latine/Hispanic students reference growth mindset, neuroplasticity, and the influence of academic experiences significantly more than other race groups

Latine/Hispanic American students exhibited the highest proportion of growth mindset responses compared to other racial and ethnic subgroups. Similarly, the Latine/Hispanic group also demonstrated a higher proportion of references to academic experiences influencing their mindset beliefs and to neuroplasticity in response to “Do you think someone can change their intelligence level? Please describe how you came to your belief, if it has ever changed, and why it changed if applicable.” (Limeri, Choe, et al., 2020).

Latine/Hispanic students demonstrated particularly strong alignment with growth mindset beliefs, with 93% expressing growth mindset themes in their responses—significantly higher than every other racial or ethnic subgroup (Black/African American/Afro-Caribbean (73.33%; adjusted ****p < 0.0001), Multiracial (46.67%; adjusted ****p < 0.0001), East Asian/Asian American (82.50%; adjusted ****p < 0.0001), South Asian/Indian American (78.85%; adjusted ****p < 0.0001), and White/Non-Hispanic/European American (61.96%; adjusted ****p < 0.0001); Middle Eastern/Arab American (83.33%; adjusted p = 0.0485). The Latine/Hispanic group also referenced neuroplasticity concepts most frequently (15%) and statistically significantly in comparison to all racial subgroups (adjusted ****p < 0.0001), often connecting these scientific principles to their academic experiences. Again, statistically significant in comparison to all subgroups (adjusted ****p < 0.0001), 78% of Latine/Hispanic students explicitly tied their mindset development to overcoming academic challenges or access to resources throughout their educational career.

Thematic differences emerged in definitions of intelligence: White students were more likely to frame intelligence as static knowledge (27% vs. 15% Latine; adjusted p < 0.0001). However, all subgroups emphasized malleable abilities (89% Latine, 80% Black, 67% Multiracial, 83% Middle Eastern, 80% East Asian, 75% South Asian, and 79% White students). These qualitative patterns align with our quantitative results showing overall strong growth mindset among all participants, but the strongest alignment among PEERs, particularly Latine/Hispanic students.

## DISCUSSION

We examined variations in mindset among neuroscience students from diverse demographics, including race/ethnicity, gender, generation status, major, and academic year. Through quantitative and qualitative analysis, we found that students across demographic groups generally aligned with growth mindset principles. Specifically, we found an increased growth mindset in PEERs—driven by the Latine/Hispanic student population—relative to White, Non-Hispanic/European American and Black, African American, Afro-Caribbean individuals.

Our findings somewhat align with Hwang et al.’s (2019) mathematics study, where dominant, privileged demographics exhibited stronger fixed mindset beliefs. Our results deviate from biology/physics literature, where historically excluded groups demonstrated lower growth mindsets and typically benefited most from interventions (Canning & Limeri, 2023). Taken together, our results and prior work suggest that disciplinary context matters and may influence mindset variations across student groups and the potential impact of interventions. Perhaps, Neuroscience’s neuroplasticity focus cultivates growth mindsets in specific populations. For example, required psychology and neuroscience introductory courses may explicitly cover growth mindset and provide students with a neurobiological understanding of how the brain can change and develop new neural connections, a key tenet of growth mindset. Importantly, this doesn’t negate or minimize growth mindset’s value as an educational equalizer but highlights that baseline mindsets may vary substantially by field and subgroup.

While we observed significant variation in mindset scores and responses across racial and ethnic groups, this effect did not extend to the other demographic groups examined. This finding contrasts with previous research, which identifies women as a historically excluded group that often experiences lower self-efficacy and a stronger fixed mindset in physics (Malespina et al., 2022). In our study, women comprised approximately 60% of the surveyed population. In the context of the large public R1 institution we investigated, over half of the student population at the university and within the neuroscience major identifies as female, and most instructors in the institution’s neuroscience department are also women (Myers-Miller, 2024). This demographic composition may have lessened female students’ susceptibility to structural barriers, fostering a stronger sense of belonging and reducing gender-based mindset disparities seen in other fields, such as physics, where men significantly outnumber women (Malespina et al., 2022; Myers-Miller, 2024).

Our findings reveal how disciplinary context may and how demographics do shape growth mindset beliefs, urging the continuation of DEI initiatives and research to promote educational equity, especially in STEM fields where the effects of historical exclusion persist.

### Limitations

Several limitations exist in this study. This cross-sectional design prevented establishing causal relationships or assessing whether specific neuroscience courses influenced students’ beliefs. Self-reported data may be subject to social desirability bias, particularly given students’ likely familiarity with growth mindset concepts in neuroscience curricula. Additionally, our deductive and inductive coding approaches may introduce confirmation bias. We used Copilot AI for independent code validation, though AI limitations with nuanced responses required human oversight for complex codes.

Unequal sample sizes across racial subgroups limit generalizability and statistical power. The single-select demographic format may have inadequately captured multiracial identities, as participants with diverse backgrounds were grouped together despite potentially different experiences. We did not assess socioeconomic status, which may better indicate resource access than generation status. Prior studies have assessed SES through parental education, household income, and school-level indicators such as free/reduced lunch eligibility and financial aid (Burnette et al., 2022; Macnamara & Burgoyne, 2022).

### Future Directions

Future studies should investigate the influence of the neuroscience curriculum on students’ growth mindset using a pre- and post-course model throughout multiple points in a neuroscience curriculum and compare those findings to a similar model within other STEM disciplines. Due to our focus on investigating mindset variation within neuroscience, most participants were neuroscience majors, and all were recruited from neuroscience courses. As a result, this study was unable to determine whether neuroscience majors and non-neuroscience majors held significantly different views on mindset. Given the strong growth mindset trends observed in this population, further research should explore whether neuroscience students differ from students in other STEM disciplines that do not emphasize the brain’s capacity to change.

Additionally, future studies should consider implementing a longitudinal method to track how mindset scores may vary across academic years. Since first-year students have not completed their major curriculum, they may have a different perspective than later-year students who have garnered experience with rigorous coursework and have persisted through challenges. A longitudinal study could follow first-year students through their years to track major changes, such as shifts from neuroscience to other disciplines or careers outside of STEM, as well as changes in mindset. This approach would provide insight into how academic success and failure, alongside growth mindset, influence retention rates of historically excluded individuals in STEM, particularly within the neuroscience major.

Future mindset studies should consider using the Undergraduate Lay Theories of Abilities (ULTrA) survey, as our qualitative responses revealed that participants with similar quantitative levels of growth mindset varied in their beliefs about their abilities (Limeri et al., 2023). Lay theories offer a comprehensive approach to understanding students’ academic outlooks by encompassing beliefs about the potential to improve intelligence (mindset), who has the capacity for excellence in a field (universality), and whether achieving excellence requires innate intellectual talent (brilliance). Given the complexity of our qualitative responses, examining mindset, brilliance beliefs, and universality beliefs as distinct yet interrelated constructs may be valuable. Limeri et al. (2023) found that these beliefs are conceptually and empirically distinct, each uniquely predicting student outcomes. Students can simultaneously hold both growth and fixed mindset beliefs, as well as universal and non-universal beliefs.

While our findings show generally strong growth mindsets in neuroscience students, targeted interventions like those in Hecht et al. (2022) could still prove valuable. Interventions can actively sustain such beliefs during academic challenges and extend benefits to subgroups with comparatively lower growth mindsets—more than passive curricular exposure alone. Some individual participants expressed strong fixed mindset beliefs despite exposure to growth mindset concepts through the neuroscience curriculum. The Framework for Implementation of Mindset Interventions (FIMI) offers a promising approach and addresses the variability often observed in mindset research (Burnette et al., 2022). FIMI emphasizes core messaging about neuroplasticity, evidence-based strategy instruction, and contextual adaptation; elements that could further strengthen growth mindsets within neuroscience curricula where these principles are already embedded.

### Address correspondence to:

Dr. Sabrina E. Robertson, Department of Psychology and Neuroscience, 230 Davie Hall, University of North Carolina at Chapel Hill, Chapel Hill, NC 27514. Email: sabrinae@email.unc.edu

## Supplementary Material

Supplementary Materials
