# Supplementary material for: Mindset Variations Among Undergraduate Neuroscience Students
Source: J Undergrad Neurosci Educ. 2025 Dec 31;24(1):83–97. doi: 10.59390/001c.154215 (PMC13127675; doi:10.59390/001c.154215)
Supplement: Supplementary Materials [file junejournal_2025_24_1_154215_321700.pdf]

SUPPLEMENTAL MATERIALS

Supplemental Table 1. Survey Questions

Defining Intelligence and Failure	
Question 1	How do you define intelligence? (Limeri et. al. 2020b)
Question 2	How does intelligence relate to academic performance? (Limeri et. al. 2020b)
Question 3	Do you think someone can change their intelligence level? (Limeri et. al. 2020b)
Question 4	For the above question, Please describe how you came to your belief, if it has ever changed and why it changed if applicable. (Limeri et. al. 2020b)
Question 5	How do you tend to react to setbacks, struggles, or failures? (Limeri et. al. 2020b)
**Growth Mindset Survey Questions**	
Question 6.1 (negatively coded)	You have a certain amount of intelligence and you can not do much about it. (Dweck, 2006)
Question 6.2 (negatively coded)	Your intelligence is something about you that you can not change very much. (Dweck, 2006)
Question 6.3 (negatively coded)	You can learn new things, but you can not really change your basic intelligence. (Dweck, 2006)
Question 6.4 (negatively coded)	You have to be born with the ability to be good at neuroscience/STEM subjects. (Dweck, 2006)
Question 6.5 (positively coded)	I can change my intelligence in neuroscience quite a lot by working hard. (Malespina et. al 2022)
Question 6.6 (negatively coded)	Only a few specially qualified people are capable of really understanding neuroscience. (Malespina et. al 2022)
Question 6.7 (negatively coded)	To really excel in STEM, people need to have a natural ability. (Malespina et. al 2022)
Question 6.8 (negatively coded)	Even if I were to spend a lot of time working on difficult STEM problems, I cannot develop my intelligence further. (Malespina et. al 2022)
**Demographics**	
Question 15	What is your current age in years?
Question 16	What is your affiliation with UNC-Chapel Hill? - Selected Choice<br>1st year undergraduate student<br>2nd year undergraduate<br>3rd year undergraduate<br>4th year undergraduate<br>5th year undergraduate<br>6th year undergraduate<br>Graduate student

	Not listed. Please fill in here:<br>Prefer not to answer
Question 17	What is your current GPA (please list N/A if you do not have a GPA)?
Question 18	Select the racial category with which you most identify. - multiple choice<br>Black, African American, Afro-Caribbean<br>East Asian / Asian American<br>Hawaiian / Pacific Islander<br>Latine / Hispanic American<br>Middle Eastern / Arab American<br>Multiracial<br>Native American / Alaskan Native<br>South Asian / Indian American<br>White, Non-Hispanic / European American<br>Not listed. Please fill in here:<br>Prefer not to answer
Question 19	Select the term(s) with which you most identify, even if it is the same as above. - multiple choice<br>Agender<br>Cisgender man<br>Cisgender woman<br>Gender non-conforming<br>Gender variant<br>Genderqueer<br>Intersex<br>Non-binary<br>Transgender man<br>Transgender woman<br>Not listed. Please fill in here:<br>Prefer not to answer
Question 20	Are you a first-generation college student (i.e., neither of your parents obtained a college degree)?
Question 21	Please check all of the Neuroscience courses listed below that you have already completed or are currently taking. - select all that apply
Question 22	Approximately how many Neuroscience courses (i.e., courses with the NSCI designation) have you completed at UNC?
Question 23	Have you worked in a research laboratory (e.g., as a research assistant, lab manager, project manager, volunteer)?
Question 24	What are your post-graduate plans? (Note: STEM = Science, Technology, Engineering, and Math, and includes health care) - Selected Choice<br>Graduate or professional studies in a STEM field<br>Graduate or professional studies in a non-STEM field<br>Employment in a STEM field<br>Employment in a non-STEM field<br>Other: Please explain__________<br>Prefer not to respond
Question 25	Did you take a Thrive course at UNC?
Question 26	How many credits are you taking this semester? Next semester?

Question 27	In what field(s) is/are your major(s)? (Limeri et al. 2020b) - multiple choice<br>Chemistry<br>Life Sciences<br>Environmental / Earth / Agricultural Sciences<br>Physics<br>Engineering<br>Mathematics<br>Psychology<br>Neuroscience<br>Humanities<br>Other major: fill in<br>Undecided
Question 28	For Neuroscience majors, do you plan on completing your degree in neuroscience or plan on changing your major? (Respond N/A is not applicable)
Question 29	Have you changed your major?
Question 30	What did you change your major from, and what did you change your major to if applicable?

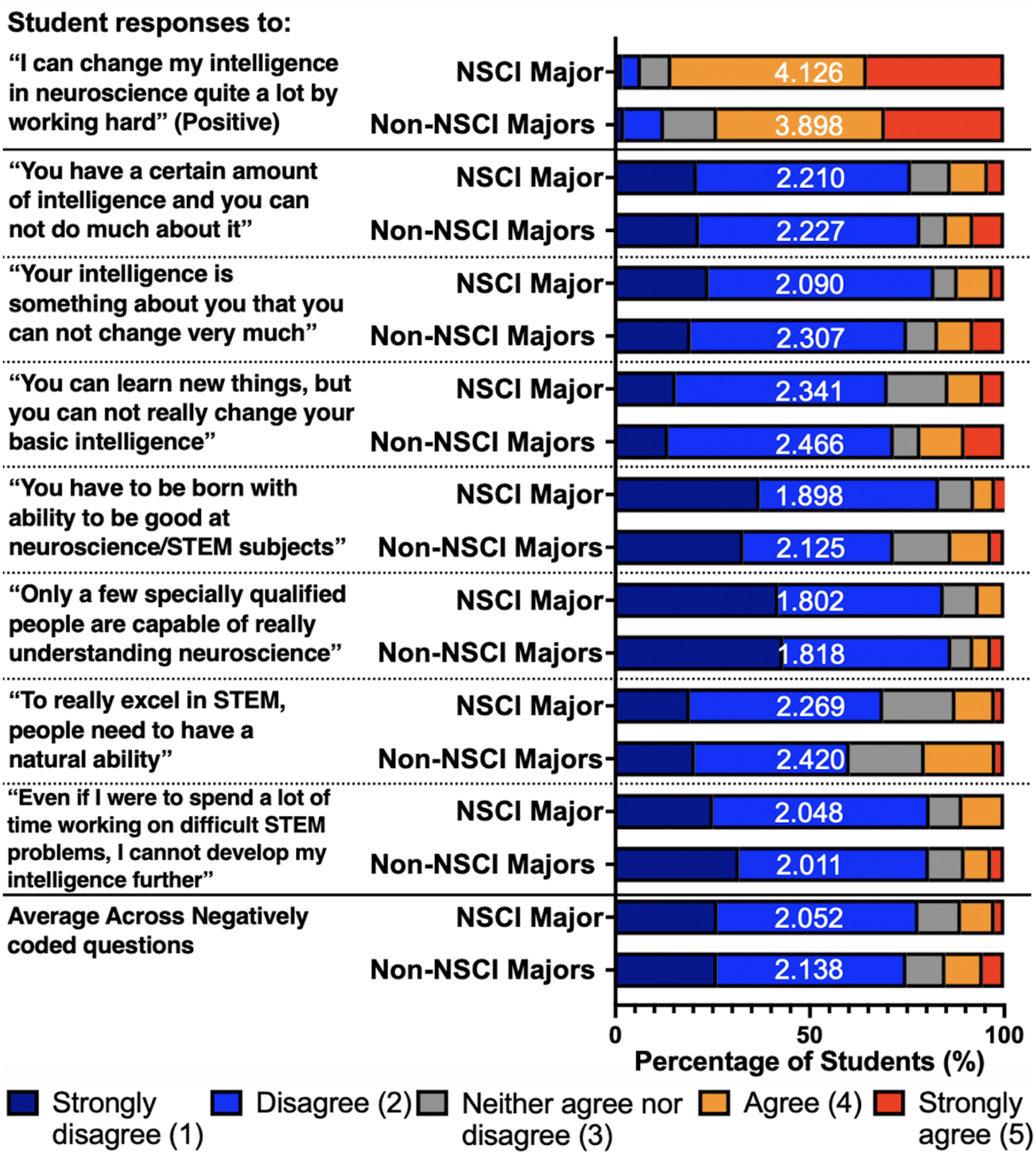

Supplemental Figure 1. Neuroscience Majors and Non-Neuroscience Majors Exhibit Similar Mindset Scores. The bar graphs display composite mindset scores for Neuroscience (NSCI) majors (n = 167) and non-NSCI majors (n = 88). The average mindset score for each group is displayed within each bar. For negatively coded questions, higher scores (closer to 5) indicate stronger agreement with fixed mindset statements (e.g., "You can't change your intelligence"), while lower scores (closer to 1) reflect stronger agreement with growth mindset statements. The positively coded question ("I can change my intelligence...by working hard") is scored inversely, with higher scores (closer to 5) indicating stronger growth mindset agreement. Mann-Whitney U tests revealed no statistically significant differences between groups across all questions and composite averages (all $p > 0.05$). However, the positively coded statement showed a trend toward significance ($p = 0.0992$), with NSCI majors ($M = 4.126$) expressing stronger growth mindset agreement than non-NSCI majors ($M = 3.898$).

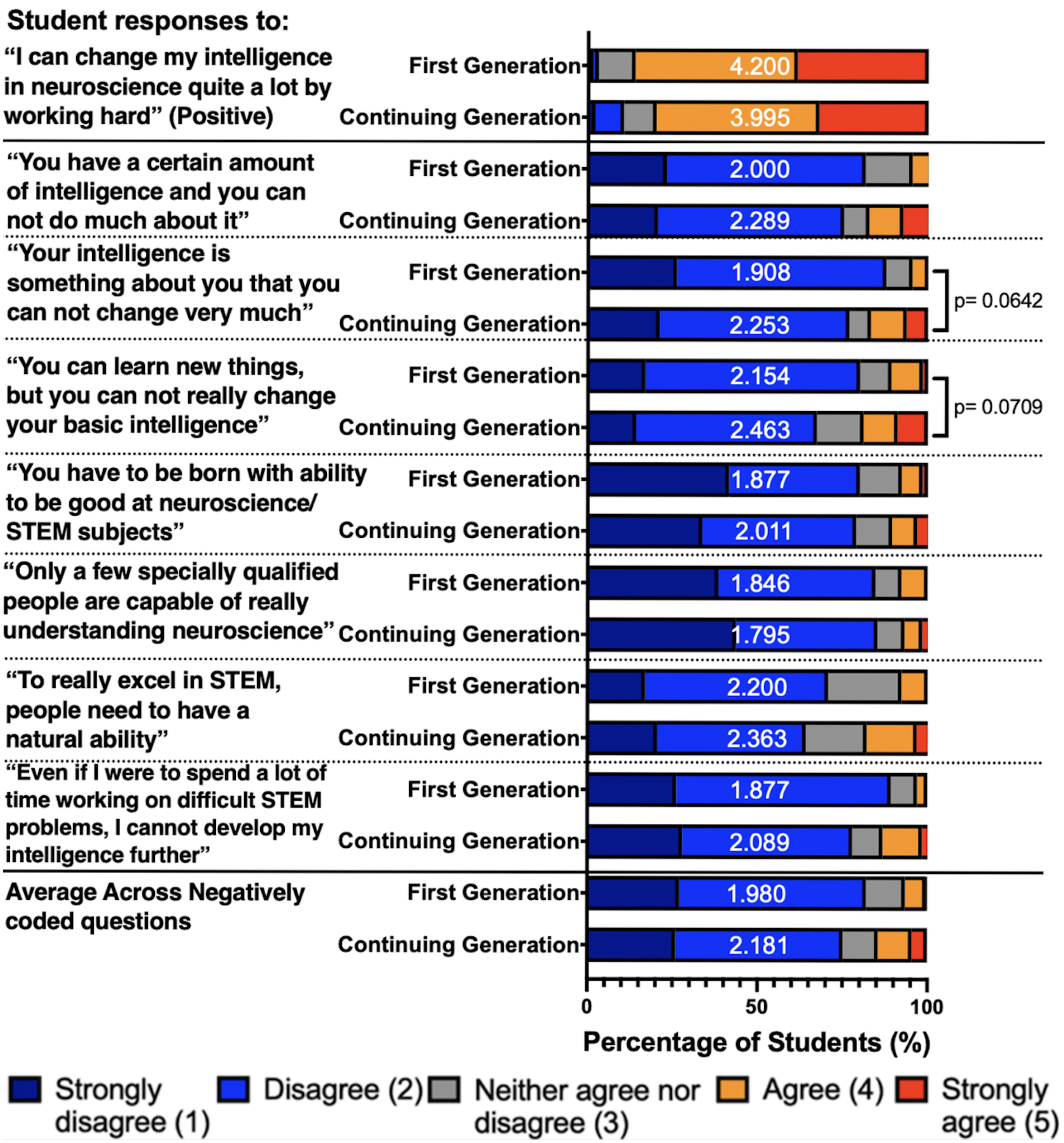

Supplemental Figure 2. First-Generation and Continuing-Generation Students Exhibit Similar Mindset Scores. The bar graphs display composite mindset scores for first-generation (n = 65) and continuing-generation (n = 190) students. The average mindset score for each group is displayed within each bar. For negatively coded questions, higher scores (closer to 5) indicate stronger agreement with fixed mindset statements (e.g., "You can't change your intelligence"), while lower scores (closer to 1) reflect stronger agreement with growth mindset statements. The positively coded question ("I can change my intelligence...by working hard") is scored inversely, with higher scores (closer to 5) indicating stronger growth mindset agreement. Mann-Whitney U tests revealed two questions nearing statistical significance: "You can learn new things, but you cannot really change your basic intelligence" (p = 0.0642) and "You have to be born with the ability to be good at neuroscience/STEM subjects" (p = 0.0709). First-generation students (M = 2.154 and M = 1.877, respectively) expressed stronger growth mindset agreement compared to continuing-generation students (M = 2.463 and M = 2.011, respectively). However, no significant differences were observed between groups across all other questions and composite averages (all $p > 0.05$)

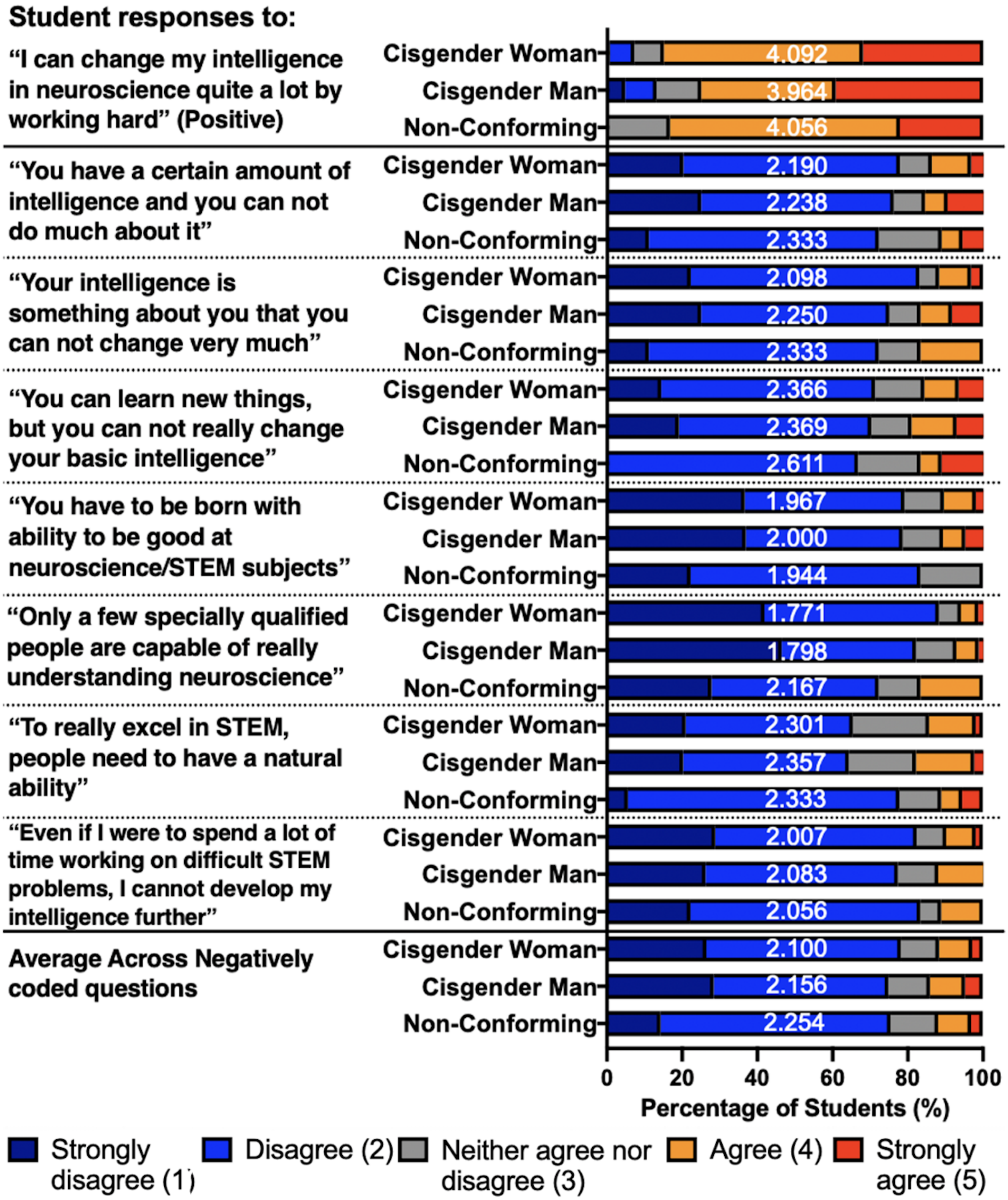

Supplemental Figure 3. Mindset Scores Are Similar across Gender Identity Groups. Bar graphs display composite mindset scores for cisgender women (*n* = 153), cisgender men (*n* = 84), and gender non-conforming students (*n* = 18). The average mindset score for each group is displayed within each bar. For negatively coded questions, higher scores (closer to 5) indicate stronger agreement with fixed mindset statements (e.g., "You can't change your intelligence"), while lower scores (closer to 1) reflect stronger agreement with growth mindset statements. The positively coded question ("I can change my intelligence...by working hard") is scored inversely, with higher scores (closer to 5) indicating stronger growth mindset agreement. A one-way ANOVA with Tukey's post hoc test revealed no statistically significant differences between any groups across all questions and composite averages (all $p > 0.05$).

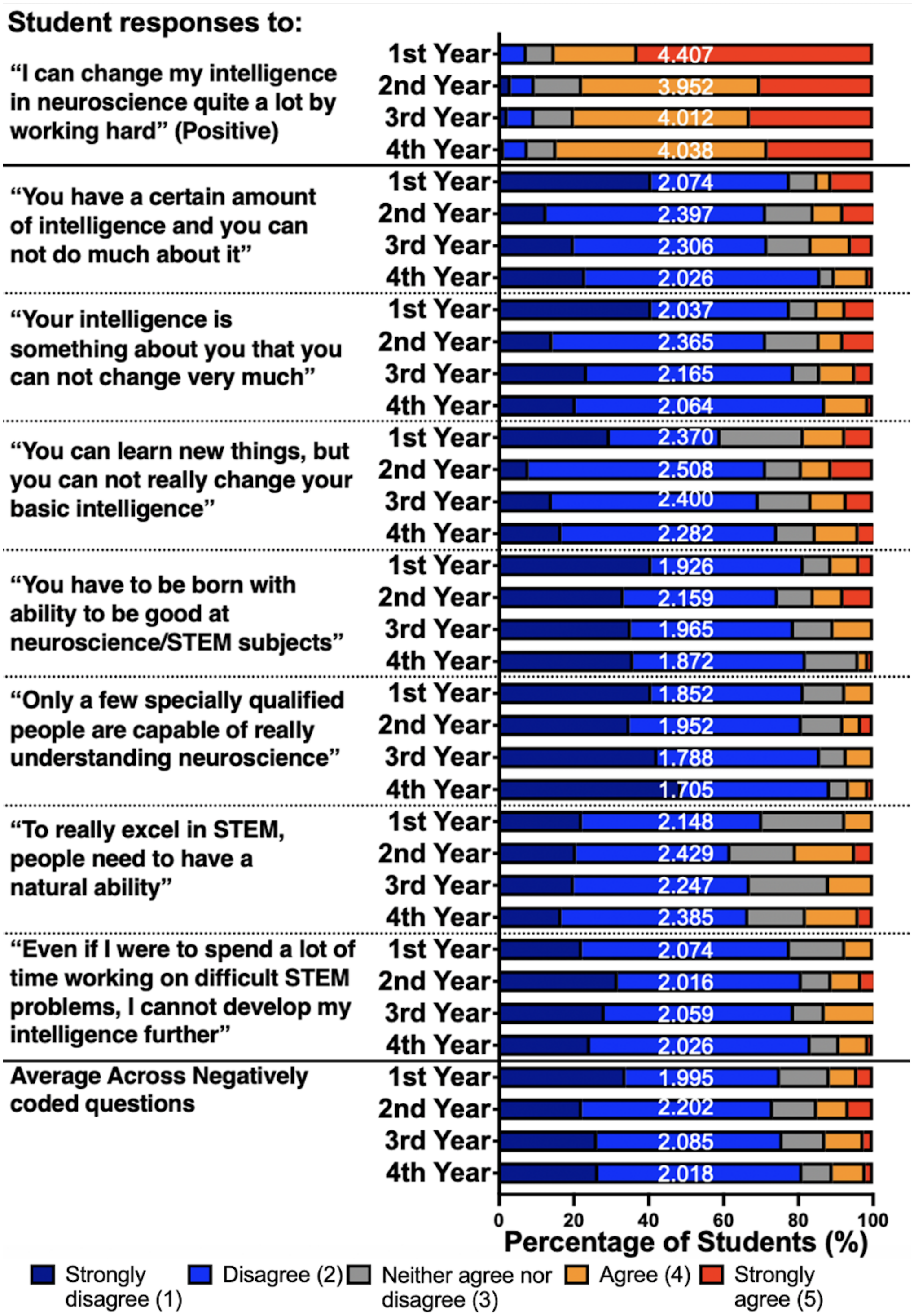

Supplemental Figure 4. Mindset Scores Are Consistent Across Academic Year. Bar graphs display composite mindset scores for 1st-year (n = 27, 2nd-year (n = 63), 3rd-year (n = 85), and 4th-year (n = 78) students. The average mindset score for each group is displayed within each bar. For negatively coded questions, higher scores (closer to 5) indicate stronger agreement with fixed mindset statements (e.g., "You can't change your intelligence"), while lower scores (closer to 1) reflect stronger agreement with growth mindset statements. The positively coded question ("I can change my intelligence...by working hard") is scored inversely, with higher scores (closer to 5) indicating stronger growth mindset agreement. A one-way ANOVA with Tukey's post hoc test revealed no statistically significant differences between any year groups across all individual questions or composite averages (all $p > 0.05$).

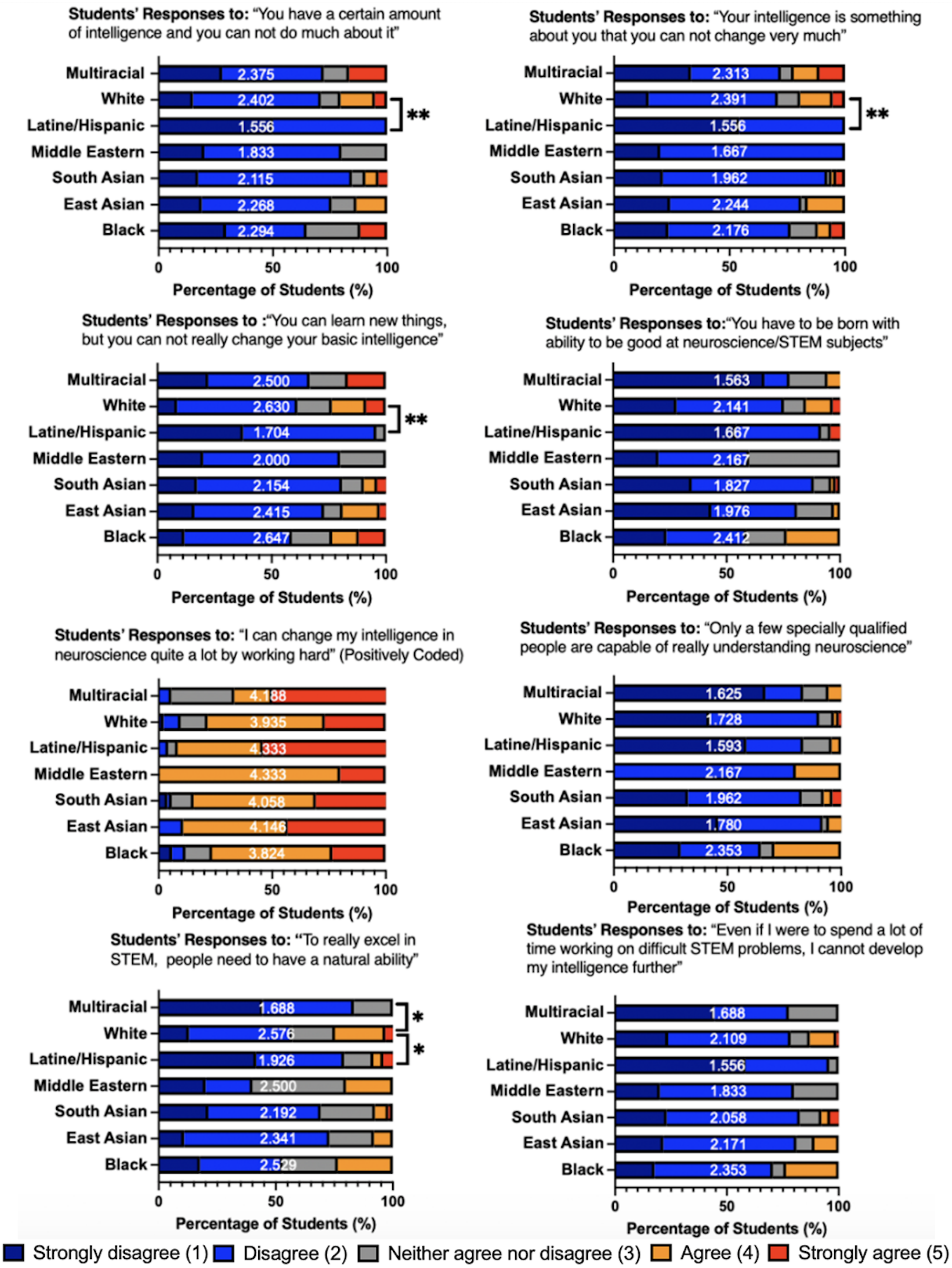
Students' Responses to: "You have a certain amount of intelligence and you can not do much about it"
Multiracial 2.375
White 2.402
Latine/Hispanic 1.556
Middle Eastern 1.833
South Asian 2.115
East Asian 2.268
Black 2.294
**
Percentage of Students (%)
Students' Responses to: "Your intelligence is something about you that you can not change very much"
Multiracial 2.313
White 2.391
Latine/Hispanic 1.556
Middle Eastern 1.667
South Asian 1.962
East Asian 2.244
Black 2.176
**
Percentage of Students (%)
Students' Responses to :"You can learn new things, but you can not really change your basic intelligence"
Multiracial 2.500
White 2.630
Latine/Hispanic 1.704
Middle Eastern 2.000
South Asian 2.154
East Asian 2.415
Black 2.647
**
Percentage of Students (%)
Students' Responses to:"You have to be born with ability to be good at neuroscience/STEM subjects"
Multiracial 1.563
White 2.141
Latine/Hispanic 1.667
Middle Eastern 2.167
South Asian 1.827
East Asian 1.976
Black 2.412
Percentage of Students (%)
Students' Responses to: "I can change my intelligence in neuroscience quite a lot by working hard" (Positively Coded)
Multiracial 4.188
White 3.935
Latine/Hispanic 4.333
Middle Eastern 4.333
South Asian 4.058
East Asian 4.146
Black 3.824
Percentage of Students (%)
Students' Responses to: "Only a few specially qualified people are capable of really understanding neuroscience"
Multiracial 1.625
White 1.728
Latine/Hispanic 1.593
Middle Eastern 2.167
South Asian 1.962
East Asian 1.780
Black 2.353
Percentage of Students (%)
Students' Responses to: "To really excel in STEM, people need to have a natural ability"
Multiracial 1.688
White 2.576
Latine/Hispanic 1.926
Middle Eastern 2.500
South Asian 2.192
East Asian 2.341
Black 2.529
*
*
Percentage of Students (%)
Students' Responses to: "Even if I were to spend a lot of time working on difficult STEM problems, I cannot develop my intelligence further"
Multiracial 1.688
White 2.109
Latine/Hispanic 1.556
Middle Eastern 1.833
South Asian 2.058
East Asian 2.171
Black 2.353
Percentage of Students (%)
0 50 100
Strongly disagree (1) Disagree (2) Neither agree nor disagree (3) Agree (4) Strongly agree (5)

Supplemental Figure 5. Race and ethnicity Subgroups mindset measures. Bar graphs display mindset responses across racial/ethnic subgroups. A one-way ANOVA with Tukey's post hoc test revealed significant differences, with Latine/Hispanic students consistently showing stronger growth mindset tendencies (lower agreement with fixed mindset statements). Compared to White students, Latine/Hispanic students reported significantly lower agreement on "You have a certain amount of intelligence and you cannot do much about it" ($M = 1.56$ vs. 2.40, $p = 0.0036$), "Your intelligence is something about you that you cannot change very much" ($M = 1.56$ vs. 2.39, $p = 0.0030$), and "You can learn new things, but you cannot really change your basic intelligence" ($M = 1.70$ vs. 2.63, $p = 0.0013$). For "To really excel in STEM, people need to have a natural ability," White students ($M = 2.58$) agreed more strongly than both Latine/Hispanic ($M = 1.93$, $p = 0.0444$) and Multiracial ($M = 1.69$, $p = 0.0172$) students.

Supplemental Table 2. *Microsoft Copilot AI Prompt Examples*

**Copilot, help me thematically code these qualitative responses.**<br>Students are answering the question: "How do you define intelligence?" Some responses are nonsensical (e.g., keyboard smashes). Each response corresponds to a participant coded as P1 through P304. I will provide the data via an Excel file.<br>Please organize your coding in a table with the following structure:<br>● Each row represents a participant code (P1 to P25).<br>● Include a column for the participant code, their racial and ethnic identities (based on the provided key), their qualitative response, and the thematic codes related to their response based on your discretion.<br>**Racial and Ethnic Identity Key:**<br>1 = Black, African American, Afro-Caribbean<br>2 = East Asian / Asian American<br>3 = Hawaiian / Pacific Islander<br>4 = Latine / Hispanic American<br>5 = Middle Eastern / Arab American<br>6 = Native American / Alaskan Native<br>7 = South Asian / Indian American<br>8 = White, Non-Hispanic / European American<br>9 = Multiracial<br>10 = Not listed<br><br>**Do you understand the task?**
**Thematic Codes:**<br>● **Knowledge**: Refers to how much knowledge someone has about a topic (an entity that someone possesses).<br>● **Ability**: Refers to the ability to learn, solve problems, or what someone can do with the knowledge they possess.<br>Please count when a student references "knowledge" or "ability" in their response and list it alongside their participant code (e.g., if P1 mentions knowledge, list "P1: 1" for knowledge).<br>**Are you ready for me to paste the first subset of responses (P1 to P25)?**
**Confirming Codes**<br>Identify the participants that indicate in their qualitative response to Question [pasted in qualitative question number and associated text] that align with [qualitative code from researcher codebook with its accompanied definition based on the literature or dictionary definition]. Give a rationalization as to why you assigned [target code] to each participant's responses.

Supplemental Table 3. *Statistically Significant Between-Group Comparisons among All Racial and Ethnic Subgroups Represented in Figure 4 for the Growth Mindset Code*

				**95% CI**	
Reference Group	***Comparison Group***	***Mean Diff.***	***Std. Error***	***Lower Bound***	***Upper Bound***
Black/African American	*East Asian/Asian American*	*-0.0917****	*0.0208*	*-0.1536*	*-0.0298*
	South Asian/Indian American	*-0.0551*	*0.0202*	*-0.1150*	*0.0048*
	White, Non-Hispanic	*0.1138*****	*0.0191*	*0.0569*	*0.1707*
	Latine/Hispanic American	*-0.1926*****	*0.0221*	*-0.2584*	*-0.1268*
	Middle Eastern/Arab American	*-0.1000**	*0.0332*	*-0.1987*	*-0.0013*
	Multiracial	*0.2667*****	*0.0251*	*0.1920*	*0.3413*
East Asian/Asian American	*South Asian/Indian American*	*0.0365*	*0.0145*	*-0.0065*	*0.0795*
	White, Non-Hispanic	*0.2054*****	*0.0130*	*0.1667*	*0.2441*
	Latine/Hispanic American	*-0.1009*****	*0.0171*	*-0.1518*	*-0.0500*
	Middle Eastern	*-0.0083*	*0.0301*	*-0.0978*	*0.0812*
	Multiracial	*0.3583*****	*0.0208*	*0.2964*	*0.4202*
South Asian/Indian American	*White, Non-Hispanic*	*0.1689*****	*0.0119*	*0.1334*	*0.2044*
	Latine/Hispanic American	*-0.1375*****	*0.0163*	*-0.1860*	*-0.0890*
	Middle Eastern	*-0.0449*	*0.0296*	*-0.1330*	*0.0433*
	Multiracial	*0.3218*****	*0.0202*	*0.2619*	*0.3817*
White, Non-Hispanic	*Latine/Hispanic American*	*-0.3064*****	*0.0151*	*-0.3511*	*-0.2616*
	Middle Eastern/Arab American	*-0.2138*****	*0.0290*	*-0.2999*	*-0.1276*
	Multiracial	*0.1529*****	*0.0191*	*0.0960*	*0.2098*
Latine/Hispanic American	*Middle Eastern/Arab American*	*0.0926**	*0.0310*	*0.0003*	*0.1848*
	Multiracial	*0.4593*****	*0.0221*	*0.3934*	*0.5251*
Middle Eastern/Arab American	*Multiracial*	*0.3667*****	*0.0332*	*0.2679*	*0.4654*

*** p < 0.05** ***** p < 0.01** ****** p < 0.001** ******* p < 0.0001***

Supplemental Table 4. *Statistically Significant Between-Group Comparisons among All Racial and Ethnic Subgroups Represented in Figure 4 for the Academic Experience Code*

				95% CI	
Reference Group	***Comparison Group***	***Mean Diff.***	***Std. Error***	***Lower Bound***	***Upper Bound***
Black/African American	*East Asian/Asian American*	*-0.0917***	*0.0236*	*-0.1618*	*-0.0215*
	South Asian/Indian American	*-0.0821***	*0.0228*	*-0.1500*	*-0.0141*
	White, Non-Hispanic	*0.1638*****	*0.0217*	*0.0992*	*0.2283*
	Latine/Hispanic American	*-0.2444*****	*0.0251*	*-0.3191*	*-0.1698*
	Middle Eastern/Arab American	*0.2000*****	*0.0376*	*0.0881*	*0.3119*
	Multiracial	*0.2667*****	*0.0285*	*0.1821*	*0.3513*
East Asian/Asian American	*South Asian/Indian American*	*0.009615*	*0.01639*	*-0.0391*	*0.0584*
	White, Non-Hispanic	*0.2554*****	*0.0148*	*0.2115*	*0.2993*
	Latine/Hispanic American	*-0.1528*****	*0.0194*	*-0.2105*	*-0.0951*
	Middle Eastern/Arab American	*0.2917*****	*0.0341*	*0.1902*	*0.3931*
	Multiracial	*0.3583*****	*0.0236*	*0.2882*	*0.4285*
South Asian/Indian American	*White, Non-Hispanic*	*0.2458*****	*0.0135*	*0.2056*	*0.2860*
	Latine/Hispanic American	*-0.1624*****	*0.0185*	*-0.2174*	*-0.1074*
	Middle Eastern/Arab American	*0.2821*****	*0.0336*	*0.1821*	*0.3820*
	Multiracial	*0.3487*****	*0.0228*	*0.2808*	*0.4166*
White, Non-Hispanic	*Latine/Hispanic American*	*-0.4082*****	*0.0171*	*-0.4589*	*-0.3575*
	Middle Eastern	*0.0362*	*0.0328*	*-0.0614*	*0.1339*
	Multiracial	*0.1029*****	*0.0217*	*0.0384*	*0.1674*
Latine/Hispanic American	*Middle Eastern/Arab American*	*0.4444*****	*0.0352*	*0.3399*	*0.5490*
	Multiracial	*0.5111*****	*0.0251*	*0.4365*	*0.5857*
Middle Eastern	*Multiracial*	*0.0667*	*0.0376*	*-0.0453*	*0.1786*

**** p < 0.05   ** p < 0.01   *** p < 0.001   **** p < 0.0001***

Supplemental Table 5.*Statistically Significant Between-Group Comparisons among All Racial and Ethnic Subgroups Represented in Figure 4 for the Neuroplasticity Code*

				95% CI	
Reference Group	***Comparison Group***	***Mean Diff.***	***Std. Error***	***Lower Bound***	***Upper Bound***
Black/African American	*East Asian/Asian American*	*-0.0333*****	*0.0028*	*-0.0415*	*-0.0252*
	South Asian/Indian American	*-0.0295*****	*0.0027*	*-0.0374*	*-0.0216*
	White, Non-Hispanic	*0.0232*****	*0.0025*	*0.0157*	*0.0307*
	Latine/Hispanic American	*-0.0815*****	*0.0029*	*-0.0902*	*-0.0728*
	Middle Eastern/Arab American	*0.0667*****	*0.0044*	*0.0536*	*0.0797*
	Multiracial	*-0.0667*****	*0.0033*	*-0.0765*	*-0.0568*
East Asian/Asian American	*South Asian/Indian American*	*0.0038*	*0.0019*	*-0.0018*	*0.0095*
	White, Non-Hispanic	*0.0565*****	*0.0017*	*0.0514*	*0.0616*
	Latine/Hispanic American	*-0.0482*****	*0.0023*	*-0.0549*	*-0.0414*
	Middle Eastern/Arab American	*0.1000*****	*0.0040*	*0.0882*	*0.1118*
	Multiracial	*-0.0333*****	*0.0028*	*-0.0415*	*-0.0252*
South Asian/Indian American	*White, Non-Hispanic*	*0.0527*****	*0.0016*	*0.0480*	*0.0574*
	Latine/Hispanic American	*-0.0520*****	*0.0022*	*-0.0584*	*-0.0456*
	Middle Eastern/Arab American	*0.0962*****	*0.0039*	*0.0845*	*0.1078*
	Multiracial	*-0.0372*****	*0.0027*	*-0.0451*	*-0.0293*
White, Non-Hispanic	*Latine/Hispanic American*	*-0.1047*****	*0.0020*	*-0.1106*	*-0.0988*
	Middle Eastern/Arab American	*0.0435*****	*0.0038*	*0.0321*	*0.0549*
	Multiracial	*-0.0899*****	*0.0025*	*-0.0974*	*-0.0823*
Latine/Hispanic American	*Middle Eastern/Arab American*	*0.1481*****	*0.0041*	*0.1359*	*0.1604*
	Multiracial	*0.0148*****	*0.0029*	*0.0061*	*0.0235*
Middle Eastern/Arab American	*Multiracial*	*-0.1333*****	*0.0044*	*-0.1464*	*-0.1203*

**** p < 0.05   ** p < 0.01   *** p < 0.001   **** p < 0.0001***

Supplemental Table 6. *Statistically Significant Between-Group Comparisons among All Racial and Ethnic Subgroups Represented in Figure 4 for the Knowledge Code*

				95% CI	
Reference Group	***Comparison Group***	***Mean Diff.***	***Std. Error***	***Lower Bound***	***Upper Bound***
Black/African American	*East Asian/Asian American*	*0.0667***	*0.0208*	*0.0047*	*0.1286*
	South Asian/Indian American	*-0.0603**	*0.0202*	*-0.1202*	*-0.0003*
	White, Non-Hispanic	*-0.0051*	*0.0192*	*-0.0621*	*0.0519*
	Latine/Hispanic American	*0.1185*****	*0.0222*	*0.0526*	*0.1844*
	Middle Eastern/Arab American	*0.1000**	*0.0332*	*0.0012*	*0.1988*
	Multiracial	*0.0667*	*0.0251*	*-0.0081*	*0.1414*
East Asian/Asian American	*South Asian/Indian American*	*-0.1269*****	*0.0145*	*-0.1700*	*-0.0839*
	White, Non-Hispanic	*-0.0717*****	*0.0130*	*-0.1105*	*-0.0330*
	Latine/Hispanic American	*0.0519**	*0.0171*	*0.0009*	*0.1028*
	Middle Eastern/Arab American	*0.0333*	*0.0301*	*-0.0563*	*0.1229*
	Multiracial	*0.0000*	*0.0208*	*-0.0620*	*0.0620*
South Asian/Indian American	*White, Non-Hispanic*	*0.0552*****	*0.0119*	*0.0197*	*0.0907*
	Latine/Hispanic American	*0.1788*****	*0.0163*	*0.1302*	*0.2273*
	Middle Eastern/Arab American	*0.1603*****	*0.0297*	*0.0720*	*0.2485*
	Multiracial	*0.1269*****	*0.0202*	*0.0669*	*0.1869*
White, Non-Hispanic	*Latine/Hispanic American*	*0.1236*****	*0.0151*	*0.0788*	*0.1684*
	Middle Eastern/Arab American	*0.1051***	*0.0290*	*0.0188*	*0.1913*
	Multiracial	*0.0717*****	*0.0192*	*0.0148*	*0.1287*
Latine/Hispanic American	*Middle Eastern/Arab American*	*-0.0185*	*0.0311*	*-0.1109*	*0.0738*
	Multiracial	*-0.0519*	*0.0222*	*-0.1178*	*0.0141*
Middle Eastern/Arab American	*Multiracial*	*-0.0333*	*0.0332*	*-0.1322*	*0.0655*

**** p < 0.05   ** p < 0.01   *** p < 0.001   **** p < 0.0001***

Supplemental Table 7. *Statistically Significant Between-Group Comparisons among All Racial and Ethnic Subgroups Represented in Figure 4 for the Ability Code*

				95% CI	
Reference Group	***Comparison Group***	***Mean Diff.***	***Std. Error***	***Lower Bound***	***Upper Bound***
Black/African American	*East Asian/Asian American*	*0.0000*	*0.0202*	*-0.0601*	*0.0601*
	South Asian/Indian American	*0.0500*	*0.0196*	*-0.0082*	*0.1082*
	White, Non-Hispanic	*0.0065*	*0.0186*	*-0.0488*	*0.0618*
	Latine/Hispanic American	*-0.0889****	*0.0215*	*-0.1528*	*-0.0250*
	Middle Eastern/Arab American	*-0.0333*	*0.0323*	*-0.1292*	*0.0625*
	Multiracial	*0.1333*****	*0.0244*	*0.0609*	*0.2058*
East Asian/Asian American	*South Asian/Indian American*	*0.0500***	*0.0140*	*0.0083*	*0.0918*
	White, Non-Hispanic	*0.0065*	*0.0126*	*-0.0311*	*0.0441*
	Latine/Hispanic American	*-0.0889*****	*0.0166*	*-0.1383*	*-0.0395*
	Middle Eastern/Arab American	*-0.0333*	*0.0292*	*-0.1202*	*0.0536*
	Multiracial	*0.1333*****	*0.0202*	*0.0732*	*0.1934*
South Asian/Indian American	*White, Non-Hispanic*	*-0.0435***	*0.0116*	*-0.0779*	*-0.0090*
	Latine/Hispanic American	*-0.1389*****	*0.0158*	*-0.1860*	*-0.0918*
	Middle Eastern/Arab American	*-0.0833*	*0.0288*	*-0.1689*	*0.0023*
	Multiracial	*0.0833*****	*0.0196*	*0.0252*	*0.1415*
White, Non-Hispanic	*Latine/Hispanic American*	*-0.0954*****	*0.0146*	*-0.1389*	*-0.0520*
	Middle Eastern/Arab American	*-0.0399*	*0.0281*	*-0.1235*	*0.0438*
	Multiracial	*0.1268*****	*0.0186*	*0.0715*	*0.1821*
Latine/Hispanic American	*Middle Eastern/Arab American*	*0.0556*	*0.0301*	*-0.0340*	*0.1451*
	Multiracial	*0.2222*****	*0.0215*	*0.1583*	*0.2861*
Middle Eastern/Arab American	*Multiracial*	*0.1667*****	*0.0323*	*0.0708*	*0.2626*

***** p < 0.05   ** p < 0.01   *** p < 0.001   **** p < 0.0001***

Supplemental Table 8. *Statistically Significant Between-Group Comparisons among All Racial and Ethnic Subgroups Represented in Figure 4 for the Work Ethic Code*

				95% CI	
Reference Group	***Comparison Group***	***Mean Diff.***	***Std. Error***	***Lower Bound***	***Upper Bound***
Black/African American	*East Asian/Asian American*	*-0.0833***	*0.0071*	*-0.1042*	*-0.0625*
	South Asian/Indian American	*-0.1167***	*0.0068*	*-0.1369*	*-0.0965*
	White, Non-Hispanic	*-0.1374***	*0.0065*	*-0.1566*	*-0.1182*
	Latine/Hispanic American	*-0.0889***	*0.0075*	*-0.1111*	*-0.0667*
	Middle Eastern/Arab American	*-0.0889***	*0.0113*	*-0.1222*	*-0.0556*
	Multiracial	*-0.1556***	*0.0085*	*-0.1807*	*-0.1304*
East Asian/Asian American	*South Asian/Indian American*	*-0.0333***	*0.0049*	*-0.0478*	*-0.0188*
	White, Non-Hispanic	*-0.0541***	*0.0044*	*-0.0671*	*-0.0410*
	Latine/Hispanic American	*-0.0056*	*0.0058*	*-0.0227*	*0.0116*
	Middle Eastern/Arab American	*-0.0056*	*0.0102*	*-0.0357*	*0.0246*
	Multiracial	*-0.0722***	*0.0071*	*-0.0931*	*-0.0514*
South Asian/Indian American	*White, Non-Hispanic*	*-0.0208***	*0.0040*	*-0.0327*	*-0.0088*
	Latine/Hispanic American	*0.0278***	*0.0055*	*0.0114*	*0.0441*
	Middle Eastern/Arab American	*0.0278*	*0.0101*	*-0.0019*	*0.0575*
	Multiracial	*-0.0389***	*0.0068*	*-0.0591*	*-0.0187*
White, Non-Hispanic	*Latine/Hispanic American*	*0.0485***	*0.0051*	*0.0335*	*0.0636*
	Middle Eastern/Arab American	*0.0485***	*0.0098*	*0.0195*	*0.0776*
	Multiracial	*-0.0181*	*0.0065*	*-0.0373*	*0.0011*
Latine/Hispanic American	*Middle Eastern/Arab American*	*0.0000*	*0.0105*	*-0.0311*	*0.0311*
	Multiracial	*-0.0667***	*0.0075*	*-0.0889*	*-0.044*
Middle Eastern/Arab American	*Multiracial*	*-0.0667***	*0.0113*	*-0.1000*	*-0.0334*

***** p < 0.05 ** ** p < 0.01 ** *** p < 0.001 ** **** p < 0.0001***

Supplemental Table 9. Additional Inductive Codes and Code Definitions.

Code Type	Code	Definition
**Inductive**	Practice (less frequent)	References to deliberate repetition or application of skills to improve performance.
	Education (less frequent)	Broader institutional or systemic influences on learning (e.g., schooling, curriculum).
	Family (less frequent)	Influences from familial expectations, support, or upbringing on mindset or learning.
	Time (less frequent)	Challenges or strategies related to time management or temporal constraints.
	Study Habits (less frequent)	Specific methods or routines used for learning or preparation.
	Health (less frequent)	Physical or mental health factors impacting learning or mindset.
	Challenges (less frequent)	Obstacles faced in academic or personal contexts that influenced mindset or performance.
	Learning (less frequent)	General references to the process of acquiring knowledge or skills.
	Resilience (less frequent)	Persistence or adaptability in the face of difficulties.
	Discipline (less frequent)	Self-regulation to complete tasks
	Consistency (less frequent)	Steady application of effort or habits over time.
	NSCI Curriculum (less frequent)	Specific references to the neuroscience curriculum's impact on mindset or learning.
	Environment (less frequent)	External settings (e.g., classroom, lab) or social contexts affecting learning.
	Skills (less frequent)	Development or application of specific competencies (e.g., problem-solving, critical thinking).
